# Multiple circulating alkaloids and saponins from intravenous Kang-Ai injection inhibit human cytochrome P450 and UDP-glucuronosyltransferase isozymes: potential drug–drug interactions

**DOI:** 10.1186/s13020-020-00349-3

**Published:** 2020-07-06

**Authors:** Zifei Qin, Mengmeng Jia, Jing Yang, Han Xing, Zhao Yin, Zhihong Yao, Xiaojian Zhang, Xinsheng Yao

**Affiliations:** 1 Department of Pharmacy, The First Affiliated Hospital of Zhengzhou University, Zhengzhou, 450052 China; 2 Henan Key Laboratory of Precision Clinical Pharmacy, Zhengzhou University, Zhengzhou, 450052 China; 3 Guangdong Province Key Laboratory of Pharmacodynamic Constituents of TCM and New Drugs Research, Jinan University, Guangzhou, 510632 China; 4 College of Pharmacy, Jinan University, Guangzhou, 510632 China

**Keywords:** Kang-Ai injection, Pharmacokinetics, Excretion, Plasma protein binding rate, Cytochrome P450, UDP-glucuronosyltransferase, Drug–drug interactions

## Abstract

**Background:**

Kang-Ai injection is widely used as an adjuvant therapy drug for many cancers, leukopenia, and chronic hepatitis B. Circulating alkaloids and saponins are believed to be responsible for therapeutic effects. However, their pharmacokinetics (PK) and excretion in vivo and the risk of drug–drug interactions (DDI) through inhibiting human cytochrome P450 (CYP) and UDP-glucuronosyltransferase (UGT) enzymes remain unclear.

**Methods:**

PK and excretion of circulating compounds were investigated in rats using a validated ultra-high-performance liquid chromatography tandem mass spectrometry (UHPLC–MS) method. Further, the inhibitory effects of nine major compounds against eleven CYP and UGT isozymes were assayed using well-accepted specific substrate for each enzyme.

**Results:**

After dosing, 9 alkaloids were found with *C*_max_ and *t*_1/2_ values of 0.17–422.70 μmol/L and 1.78–4.33 h, respectively. Additionally, 28 saponins exhibited considerable systemic exposure with *t*_1/2_ values of 0.63–7.22 h, whereas other trace saponins could be negligible or undetected. Besides, over 90% of alkaloids were excreted through hepatobiliary and renal excretion. Likewise, astragalosides and protopanaxatriol (*PPT*) *type* ginsenosides also involved in hepatobiliary and/or renal excretion. Protopanaxadiol (*PPD*) *type* ginsenosides were mainly excreted to urine. Furthermore, *PPD*-*type* ginsenosides were extensively bound (*f*_u-plasma_ approximately 1%), whereas astragalosides and *PPT*-*type* ginsenosides displayed *f*_u-plasma_ values of 12.35% and 60.23–87.36%, respectively. Moreover, matrine, oxymatrine, astragaloside IV, ginsenoside Rg1, ginsenoside Re, ginsenoside Rd, ginsenoside Rc, and ginsenoside Rb1 exhibited no inhibition or weak inhibition against several common CYP and UGT enzymes IC_50_ values between 8.81 and 92.21 μM. Through kinetic modeling, their inhibition mechanisms towards those CYP and UGT isozymes were explored with obtained K_i_ values. In vitro-in vivo extrapolation showed the inhibition of systemic clearance for CYP or UGT substrates seemed impossible due to [I]/K_i_ no more than 0.1.

**Conclusions:**

We summarized the PK behaviors, excretion characteristics and protein binding rates of circulating alkaloids, astragalosides and ginsenosides after intravenous Kang-Ai injection. Furthermore, weak inhibition or no inhibition towards these CYP and UGT activities could not trigger harmful DDI when Kang-Ai injection is co-administered with clinical drugs primarily cleared by these CYP or UGT isozymes.
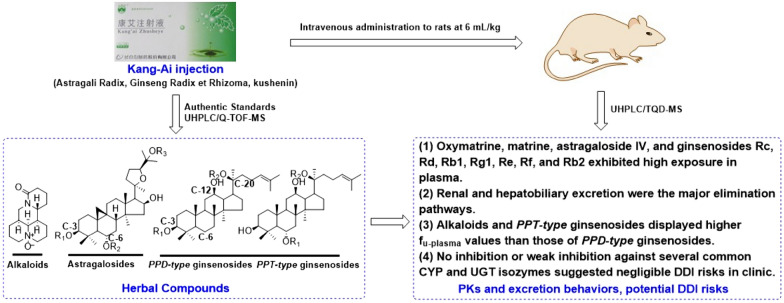

## Background

Herbal medicines are gaining popularity for the treatment of chronic and complex diseases as it works by hitting multiple molecular targets to modulate different signaling pathways [1–3]. Major advances have been made regarding their activities related their clinical indications, and more importantly in understanding their pharmacokinetics (PK) and excretion [3]. The PK profiles, just like the pharmacological activities, can be used as a “sieve” to assess the effectiveness of the individual compounds, and identify the chemical basis in an herbal injection [3–5]. Therefore, PK behaviors and excretion of circulating compounds should be first implemented in laboratory animals to reveal their systemic exposure. The corresponding PK parameters could show the ability of herbal compounds to be absorbed and transferred from the site of administration to the action targets through multiple biological barriers, and metabolic stability in vivo.

Kang-Ai injection is an herbal medicine approved in 2002 by the China Food and Drug Administration (China FDA). It consists of Astragali Radix (Huangqi), Ginseng Radix et Rhizoma (Renshen) and kushenin (Kushensu), and is indicated for many cancers, leukopenia and chronic hepatitis B [6–8]. Previously, we have finished the chemical identification and content determination of the main alkaloids (oxymatrine, matrine), astragalosides (astragaloside IV) and ginseng saponins (ginsenosides Rg1, Re) in Kang-Ai injection [9]. Despite the increasing understanding of Kang-Ai injection in chemical and pharmacological studies, the systemic exposure and excretion of its bioactive compounds after administration remain to be elucidated.

Previous PK researches of individual herb or its characteristic compounds have been carried out, which greatly facilitated the holistic PK properties of Kang-Ai injection. Oxymatrine, the most abundant herbal compound in Kang-Ai injection, occupies over 95.37% of total contents. When intravenously treated with oxymatrine at 30 mg/kg, the AUC_0–12 h_, *C*_max_, *t*_1/2_ and CL values in rat plasma were 16234.27 ng h/mL, 32847.57 ng/mL, 0.57 h and 3.10 L/h, respectively [10]. Besides, only about 19.9% of oxymatrine was reduced to matrine following intravenous infusion of 600 mg oxymatrine in healthy male volunteers [11]. In addition, systemic exposure of astragaloside IV appeared to be dose proportional over the dose ranged from 200 to 500 mL astragaloside IV (9 mg/100 mL) in healthy volunteers [12]. Furthermore, metabolism of astragaloside IV by intestinal microbiome was a crucial step in its excretion, rather than by hepatic and intestinal enzymes [13–15]. Moreover, ginsenosides Rg1, Re, and Rf were not extensively bound in plasma, and also were with substantially shorter t_1/2_ values and significantly larger systemic clearance (CL_tot,p_) than other-type ginsenosides [16]. Although several bioanalytical assays have been applied to the PK study of several circulating alkaloids and saponins, PK and excretion information about Kang-Ai injection is still limited. This also brings a huge obstacle in our understanding of the chemical basis responsible for the therapeutic effects of Kang-Ai injection.

In addition, drug–drug interaction (DDI) is significant safety concerns in clinical medication [17–19]. Inhibition or induction of human CYP or UGT isozymes could potentially adverse clinical DDI and result in metabolic disorders for endogenous components [20–22]. It would be more serious for the clinical drugs with narrow therapeutic indices, such as digoxin, warfarin, SN-38 and so on [17]. Similarly, UGT1A1 is the only one UGT isozyme in the conjugative detoxification of bilirubin [21]. Once UGT1A1 function is inhibited, there is a high risk of liver injury [21]. Considering the intravenous administration of Kang-Ai injection and abundant exposure of its circulating herbal compounds, it should be of particularly clinical significance to investigate the potential risks of DDI.

To this goal, this current report first identified the major circulating alkaloids, astragalosides, ginsenosides in rat samples after an intravenous 30 min-infusion of Kang-Ai injection at 6 mL/kg. In addition, the detailed PK behaviors, and exposure of these herbal substances in plasma were systemically carried out. Meanwhile, we also investigated the plasma binding rate assays of these circulating alkaloids and saponins. Furthermore, the excretion of these compounds in urine and bile were determined by dividing the cumulative amount excreted into urine (*Cum.A*_e-U_) and bile (*Cum.A*_e-B_) by their respect doses. Moreover, we explored the inhibitory effects and mechanism of nine major circulation compounds against several important CYP and UGT enzymes based on their widely recognized probe substrates. Taken together, the current PK and excretion information would facilitate the identification of active herbal compounds responsible for the therapeutic actions of Kang-Ai injection, and would also be helpful to inform its potential DDI in rational clinical use and achieve the optimal herbal therapy.

## Materials and methods

### Chemicals and reagents

Kang-Ai injection samples (lot numbers of 03180805, 03181004, 01180908, 01180909, 02190102, 02190103) were manufactured by Changbaishan Pharmaceutical Co., Ltd. (Jilin, China) with China FDA drug ratification number of GuoYaoZhunZiZ-20026868. Purified matrine, oxymatrine, oxysophocarpine, isoastragaloside IV, and astragaloside III, IV, V, VI were all purchased Guangzhou Fans Biotechnology Co., Ltd (Guangzhou, China). Reference standards of ginsenosides Rb1, Rb2, Rc, Rd, Re, Rf, Rg1, Rg2, Rg6, Rh1, Rh4, Rk3, F1, F2, F3, F4, and notoginsenosides R1, R2 were all obtained from Shanghai Yuanye Biotechnology Co., Ltd. (Shanghai, China). The purity of these compounds was over 98.0%.

Magnesium chloride (MgCl_2_), D-saccharic-1, 4-lactone monohydrate, alamethicin, nicotinamide adenine dinucleotide phosphate (NADPH), uridine 5′-diphospho-glucuronosyltransferase (UDPGA) were all obtained from Sigma-Aldrich (St. Louis, MO, USA). 4-hydroxytolbutamide, 4-hydroxymephenytoin, 4-methylumbelliferone (4-MU), 6α-hydroxy-paclitaxel, 6-hydroxychlorzoxazone, 7-hydroxycoumarin, β-estradiol, bupropion, coumarin, chlorzoxazone, hydroxybupropion, mephenytoin, nifedipine, oxidized nifedipine, phenacetin, paracetamol, paclitaxel, propofol and tolbutamide were all acquired from Aladdin Chemicals (Shanghai, China). 4-MU-glucuronide, β-estradiol-3-*O*-glucuronide and propofol-*O*-glucuronide were all obtained from Toronto Research Chemicals (North York, ON, Canada). Recombinant CYP1A2 (Cat. No. 456203), CYP2A6 (Cat. No. 456254), CYP2B6 (Cat. No. 456255), CYP2C8 (Cat. No. 456252), CYP2C9 (Cat. No. 456258), CYP2C19 (Cat. No. 456259), CYP2E1 (Cat. No. 456206), CYP3A4 (Cat. No. 456202), and expressed UGT1A1 (Cat. No. 456411), UGT1A9 (Cat. No. 456419), UGT2B7 (Cat. No. 456427) were all provided from Corning Biosciences (Corning, NY, USA).

LC–MS-grade water, methanol and acetonitrile were purchased from Fisher Scientific (Fair Lawn, New Jersey, USA). LC-MS grade formic acid was obtained from Sigma-Aldrich (St. Louis, USA). Other reagents were of analytical or higher grade.

### Animals

Specific pathogen free (SPF) grade male Sprague–Dawley rats (250 ± 20) g were provided by the Experimental Animal Center of Zhengzhou University (Zhengzhou, China). These rats were kept in a designated animal room at constant temperature (22 ± 2)  °C and humidity (50 ± 20)  % with 12 h of light/dark per day and free access to water and food. All protocols of animal experiments were approved in accordance with the Regulations of Experimental Animal Administration issued by the Ethics Review Committee for Animal Experimentation of Jinan University (Ethical Review NO. 20190301010). Prior to the experiments, the rats were fasted for 12 h but with free access to water. After study use, the rats were euthanized with CO_2_ gas.

### Sample preparation

Each plasma sample (50 μL) was transferred to a 1.5-mL polypropylene tube containing 50 μL of internal standard (IS) solution (including 20 nM olanzapine-d3 and 1000 nM mycophenolic acid-d3) and 50 μL of methanol. The mixture was vortex mixed for 30 s, and then 150 μL methanol was precipitated. The tubes were vortex mixed vigorously for 1 min and centrifuged at 13,800*g* for 10 min at 4 °C. Finally, 4 μL aliquots of the supernatant was injected into the UHPLC/TQD-MS system. Similarly, rat urine (50 μL) and bile (50 μL) samples were precipitated with methanol at a volumetric sample-to-methanol ratio of 1:3; after centrifugation, the resulting supernatants were applied for analysis. All the biological samples were stored at − 80 °C.

### Preparation of standard solutions and quality control samples

An appropriate amount of each authentic standard was separately dissolved with 60% methanol–water in a 10 mL volumetric flask to prepare the standard solution. Furthermore, an appropriate volume of each standard solution was transferred into a 10 mL volumetric flask to the desired concentration as the stock solution. The mixed IS solution contained olanzapine-d3 and mycophenolic acid-d3 at final concentration of 20 nM and 1000 nM, respectively. All the solutions were stored at 4 °C.

Calibration standard samples were prepared by spiking 50 μL blank plasma (or other biological samples) with 50 μL working solutions and 50 μL IS solution. Therefore, the plasma concentration ranges were 10–1000 nM for matrine (**A1**), 10–1000 nM for oxysophocarpine (**A2**), 40–4000 nM for oxymatrine (**A3**), 40–4000 nM for astragaloside IV (**B2**), 10–1000 nM for astragaloside III (**B3**), 10–1000 nM for ginsenoside Rh1 (**C1**), 10–1000 nM for notoginsenoside R2 (**C3**), 10–1000 nM for ginsenoside Rg2 (**C5**), 20–2000 nM for ginsenoside Rg1 (**C6**), 10–1000 nM for ginsenoside Rf (**C7**), 10–1000 nM for notoginsenoside R1 (**C8**), 20–2000 nM for ginsenoside Re (**C9**), 10–1000 nM for ginsenoside Rd (**D2**), 10–1000 nM for ginsenoside Rc (**D3**), 10–1000 nM for ginsenoside Rb2 (**D4**), 10–1000 nM for ginsenoside Rb1 (**D5**), respectively. Quality control (QC) samples were independently prepared in the same way at 2, 10, 40 times of LLOQ (lower limit of quantification) of each analyte. The calibration standard and QC samples were processed on each analysis day with the same procedures for plasma samples.

### Chromatographic and mass spectrometric conditions

Ultra-high-performance liquid chromatography (UHPLC) analysis was performed on a Waters Acquity UHPLC I-Class system (Manchester, UK) for the quantitative analysis of Kang-Ai injection. Chromatographic separation was achieved on an BEH C18 column (2.1 × 50 mm, 1.7 μm) with water (A) and acetonitrile (**B**) (both including 0.1% formic acid) at a flow rate of 0.45 mL/min with column temperature of 35 °C. The gradient flow profile was optimized as follows: 10% **B** from 0 to 0.5 min, 10–20% **B** from 0.5 to 1.5 min, maintaining 20% **B** from 1.5 to 4.5 min, 20–30% **B** from 4.5 to 5.5 min, 30–35% **B** from 5.5 to 7.5 min, 35–90% **B** from 7.5 to 9.5 min, keeping 90% **B** from 9.5 to 10.5 min, 90–10% **B** from 10.5 to 11.0 min, and maintaining 10% **B** from 11 to 12 min.

UHPLC system was coupled to a triple quadrupole mass spectrometer (Waters Xevo TQD, Waters, Manchester, UK). The detailed mass spectrometers were adjusted as follows: capillary voltage, 3.5 kV (ESI+) or 1.5 kV (ESI−); cone voltage, 50 V (ESI+) or 50 V (ESI−); source temperature, 350 °C; desolvation gas flow, 650 L/h; The mass spectrometer was performed in the multiple reaction monitoring mode (MRM) using both positive and negative ionization. The quantitative parameters in MRM modes were same as our previous study. All experimental data were collected and processed using a Quanlynx software in Masslynx 4.1 platform.

### Method validation

The developed UHPLC-MS/MS method was validated according to the Guidance for Industry: bioanalytical method validation from the US FDA for specificity, linearity, LLOQ, extraction recovery, matrix effects, precision, accuracy, stability and dilution integrity [23]. The assay validation of each analyte in rat biological samples including plasma, urine and bile were all performed based on the standard specification above.

Specificity was determined by comparing the chromatograms of blank samples (from six individual rats), with dosed samples after an intravenous bolus of Kang-Ai injection, and blank samples spiked with each analyte at LLOQ and IS. The calibration curves were constructed by the peak area ratios of the analytes to IS (Y), *versus* respective sample concentrations (X) applying a weighted (1/*x*^2^) least squares linear regression analysis. LLOQ was determined based on the lowest concentrations for which acceptable linearity, accuracy and precision were demonstrated. The accuracies (the relative error, RE) and inter/intra-day precisions (the relative standard deviation, RSD) of the assay were evaluated by determining six replicates of QC samples (at low, middle, and high concentrations) on three consecutive days.

Extraction recoveries (ER) and matrix effects (ME) were evaluated by a published experimental protocol [24]. The response of extracted QC sample was defined as A1. In addition, A2 referred to the corresponding standard solutions added into the post-extracted supernatant from biological matrix, while A3 corresponded to the responses of each analyte at three QC levels. The ER and ME values were calculated as follows: ER % = A1/A2 × 100%, and ME % = A2/A3 × 100%.

Stability tests were investigated at three QC levels under different conditions, including short-term stability (8 h exposure at room temperature), autosampler storage stability of the methanol-treated samples (at 8 °C for 18 h), freeze/thaw stability (three − 80 °C ↔ 23 °C cycles), long-term stability (14 days storage at − 80 °C). The dilution integrity was investigated by analyzing six replicate samples with each analyte, and each analyte were diluted 10-fold with blank rat matrix. Furthermore, the diluted samples were analyzed using freshly prepared calibration curve to calculate RE and RSD, which should be no more than ± 15% as criterion.

### In vivo rat studies

The rats in this study all received a single 30-min intravenous infusion of Kang-Ai injection via the tail veins using TYD01-02 infusion pumps (Lead Fluid, Baoding, China). The dose was derived from the label daily dose of Kang-Ai injection (60 mL/person, once daily) according to dose normalization by body surface area [25]. For plasma pharmacokinetic studies, the rats were randomly received a single intravenous 30 min-bolus dose at 6 mL/kg. Blood samples (100 μL) were collected from the rats’ external jugular veins into heparinized polypropylene tubes at 5, 15, 30, 45 min, and 1, 1.5, 2, 3, 4, 6, 8, 12 h. After gently shaking for 10 s, the blood samples were centrifuged at 13000 *g* for 10 min to yield plasma samples, which were kept frozen at − 80 °C until analysis.

For urine sampling, the rats were housed singly in metabolic cages. And then, the urine samples were collected at 0–4, 4–8 and 8–12 h after starting infusion and were weighed. Likewise, bile samples were also collected at 0–3, 3–6, 6–9 and 9–12 h after starting infusion and were weighed. The urine and bile collection tubes were all frozen at − 80 °C.

### Protein binding assay

Binding of circulating alkaloids and saponins to rat plasma was measured by equilibrium dialysis using Spectra/Por 2 dialysis membranes (molecular weight cutoff, 12–14 kDa; Rancho Dominguez, CA, USA) as described [26]. After equilibrating for 24 h at 55 rpm and 37 °C, the dialysate and the plasma were sampled for analysis. In brief, the concentrations of each analyte in dialysate and rat plasma samples were determined for analysis after equilibrating at 55 rpm and 37 °C for 24 h. The concentrations of tested matrine, oxysophocarpine and oxymatrine were 2 μmol/L, while those of astragaloside IV, III, and ginsenosides Rh1, Rg2, Rg1, Rf, Re, Rd, Rc, Rb1, Rb2, notoginsenosides R1, R2, were 4 μmol/L. The unbound fraction (*f*_u-plasma_) in rat plasma was calculated as follow: *f*_u-plasma_ = *C*_d_/*C*_p_ × 100%, where the *C*_d_ and *C*_p_ values were the concentrations of each analyte in the dialysate sample and in the post-dialysis plasma sample, respectively.

### Incubation systems

Phase I incubation system (100 μL) contained Tris–HCl buffer (50 mM, pH = 7.4), MgCl_2_ (5 mM), each CYP isozyme, specific substrates, circulating compounds and NADPH (1 mM) as prescribed previously [18]. After incubation (37 °C, 60 min), the reaction was terminated by ice-cold acetonitrile (100 μL), following by centrifugation at 13,800*g* for 10 min. The supernatant (8 μL) was acquired for ultra-high-performance liquid chromatography (UHPLC) system (Waters, Manchester, UK) analysis.

For glucuronidation assays, the incubation system (100 μL) contained Tris–HCl buffer (50 mM, pH = 7.4), alamethicin (22 μg/mL), D-saccharic-1, 4-lactone (4.4 mM), each UGT enzyme, specific substrates, circulating compounds and UDPGA (3.5 mM) as described recently [18]. The reaction was terminated by ice-cold acetonitrile (100 μL) after incubation at 37 °C for 60 min. After centrifugation (13,800*g*, 10 min), the supernatant (8 μL) was obtained for determination by UHPLC system.

### Analysis of inhibitory effects

In this study, phenacetin (100 μM), coumarin (100 μM), bupropion (100 μM), paclitaxel (60 μM), tolbutamide (200 μM), mephenytoin (100 μM), chlorzoxazone (200 μM) and nifedipine (40 μM) have been used as the probe substrates for CYP1A2, 2A6, 2B6, 2C8, 2C9, 2C19, 2E1 and 3A4, respectively [27]. After optimization of incubation conditions (Additional file 1: Fig. S1), the substrates were incubated with each CYP isozyme at different protein concentrations (0.05 mg/mL for CYP1A2 and 3A4; 0.1 mg/mL for CYP2A6, 2B6, 2C8, 2C9, 2C19 and 2E1) in the absence (control) and presence of different mixture or herbal compounds (Kang-Ai injection and samples that Kang-Ai injection was diluted 10 times; 1, 10, and 100 μM for matrine and oxymatrine; 0.1, 1, 10 and 100 μM for astragalosides and ginsenosides).

Similarly, β-estradiol (60 μM), propofol (40 μM) and 4-MU (350 μM) were typically used as the substrates for UGT1A1, 1A9 and 2B7, respectively [28]. After optimization of incubation conditions (Additional file 1: Fig. S2), the protein concentrations for UGT1A1, 1A9, and 2B7 were 0.125, 0.05, and 0.05 mg/mL, respectively. Kang-Ai injection and samples that Kang-Ai injection was diluted 10 times, were both used to investigate the inhibitory effects against UGT1A1, 1A9 and 2B7. In addition, the concentrations were 1, 10 and 100 μM for matrine and oxymatrine, and 0.1, 1, 10 and 100 μM for astragalosides and ginsenosides based on the detailed plasma concentration after intravenous Kang-Ai injection.

The half-inhibition concentration (IC_50_) values were determined by non-linear regression analysis. The inhibitory effects towards each CYP or UGT enzyme were divided into four categories as follows, potent (IC_50_ < 1 μM), moderate (1 μM < IC_50_ < 10 μM), weak (10 μM < IC_50_ < 100 μM), or no inhibition (IC_50_ > 100 μM) [18]. The inhibition mechanism towards corresponding CYP and UGT isoforms were further explored.

### Inhibition kinetic analysis

The inhibition constant (K_i_) values were obtained by multiple concentrations of substrates in the absence or presence of multiple concentrations of herbal compounds as described previously [18, 27, 28]. Competitive inhibition, noncompetitive inhibition, and mixed-type inhibition models were used to determine the K_i_ values by nonlinear regression analysis using the Eqs. (1)–(3), respectively. The model with the smallest Akaike information criterion (AIC) and Schwartz information criterion (SC) values was considered as the best model, following the obtained appropriate K_i_ values. Model fitting and parameter estimation were performed using Graphpad Prism V5 software (SanDiego, CA).

The detailed parameters for three equations were as follow. *V* is the velocity of the reaction. The [S] and [I] are the concentrations of substrate and herbal compounds, respectively. K_i_ is the constant describing the affinity between herbal compounds and the enzyme. K_m_ is the substrate concentration at half of the maximum velocity (V_max_) of the reaction. The αKi describes the affinity of herbal compounds to the complex of enzyme and substrate; When α is very large (α ≫ 1), the binding of inhibitor would prevent the binding of substrate, and the mixed inhibition model becomes identical to competitive inhibition.

1 $$ V = \frac{{V_{max} \times \left[ S \right]}}{{K_{m} \times \left( {1 + \frac{\left[ I \right]}{{K_{i} }}} \right) + \left[ S \right]}} $$

2 $$ V = \frac{{V_{max} \times \left[ S \right]}}{{\left( {K_{m} + \left[ S \right]} \right) \times (1 + \frac{\left[ I \right]}{{K_{i} }}}} $$

3 $$ V = \frac{{V_{max} \times \left[ S \right]}}{{K_{m} \times \left( {1 + \frac{\left[ I \right]}{{K_{i} }}} \right) + \left[ S \right] \times \left( {1 + \frac{\left[ I \right]}{{\alpha K_{i} }}} \right)}} $$

### Data processing

Pharmacokinetic parameters were estimated by non-compartmental analysis using WinNonlin 6.3 software (Pharsight, NC, US). Data are presented as the mean ± SD (n = 3). The area under the concentration–time curve up to the last measured point in time (AUC_0-t_) was calculated using the trapezoidal rule. The total plasma clearance (CL_tot, p_) was calculated by dividing the compound dose by the AUC_0-∞_, and the distribution volume at steady state (*V*_SS_) was estimated by multiplying the CL_tot, p_ by the mean residence time (MRT). The renal excretory clearance (CL_R_) and hepatobiliary (CL_B_) was determined by dividing the cumulative amount excreted into urine (*Cum.A*_e-U_) and bile (*Cum.A*_e-B_) by the plasma AUC_0-∞_, respectively. The fractions of dose excreted into urine (*f*_e-U_) and the fractions of dose excreted into bile (*f*_e-B_) were established using the relationship *Cum.A*_e-U_/Dose and C*um.A*_e-B_/Dose, respectively. Mean differences between treatment and control groups were analyzed by two-tailed unpaired Student’s t test by Graphpad Prism V5 software (SanDiego, CA). The level of significance was set at p < 0.05 (∗), *p* < 0.01 (∗∗) or *p* < 0.001 (∗∗∗).

## Results

### Xenobiotics detected in rat samples

After dosing Kang-Ai injection to rats (6 mL/kg), a total of 9 circulating alkaloids, 6 astragalosides and 22 ginsenosides were detected and characterized in rat samples (Additional file 1: Fig. S3A and Table S1). Based on their individual content level from Kang-Ai injection [9], these compounds were graded into five dose levels (Additional file 1: Fig. S3B and Table S1): Level I was over 1000 μg/mL for only oxymatrine (**A3**); Level II was between 100 and 1000 μg/mL for ginsenosides Rg1 (**C6**) and Re (**C9**); Level III was ranged from 10 to 100 μg/mL for matrine (**A1**), astragaloside IV (**B2**), notoginsenoside R2 (**C3**), ginsenoside Rg2 (**C5**), Rf (**C7**) and Rc (**D3**); Level IV was from 1 to 10 μg/mL for astragaloside III (**B3**), VI (**B4**), V (**B5**), notoginsenoside R1 (**C8**), ginsenoside Rh1 (**C1**), Rd (**D2**), Rb2 (**D4**), Rb1 (**D5**), Rh4 (**E2**), Rg6 (**E3**), F4 (**E4**); Level V was all less than 1 μg/mL for oxysophocarpine (**A2**), isoastragaloside IV (**B1**), ginsenoside F1 (**C2**), F3 (**C4**), F2 (**D1**), Rk3 (**E1**).

### Method validation

The typical MRM chromatograms obtained from blank plasma, blank plasma spiked with the analyte (LLOQ), and dosed rat plasma after intravenous Kang-Ai injection (30 min) were presented in Additional file 1: Fig. S4. As seen from MRM chromatograms, there were no endogenous interference for each analyte and two IS compounds, which suggested that the specificity of the quantitative method was satisfactory.

Additional file 1: Table S2 summarized the mean values of linear regression equation of the sixteen compounds. The correlation coefficients (*r*^2^) were ranged from 0.9900 to 0.9994, which exhibited good linearity. The LLOQs of each circulation compound were between 5 and 40 nM, with the accuracies and precisions less than 20%, which were enough for the pharmacokinetics study of sixteen analytes following a 30-min infusion of Kang-Ai injection to rats.

The results of the intra- and inter-day precision and accuracy of all analytes and three QC samples were displayed in Additional file 1: Table S3. The intra-day and inter-day precision (RSD) of sixteen analytes were within the range from 2.35 to 12.64%, whereas the accuracy (RE) derived from three QC samples was between − 14.30 and 13.66%, all meeting the requirements. These results proved that the developed method was accurate and reproducible.

Additional file 1: Table S4 exhibited the matrix effect and extraction recovery results of all analytes and two IS. The mean matrix effects of 16 circulation compounds were in the range from 92.06 to 110.45% with RSD values less than 14.14%, and the average absolute matrix effects of olanzapine-d3 (IS-1) and mycophenolic acid-d3 (IS-2) were 100.29% and 109.12% with RSD values of 11.42% and 3.13%, respectively. Similarly, the mean extraction recoveries ranged from 82.85% to 108.98% with RSD values no more than 14.73% at three QC levels, whereas the mean absolute recoveries of IS-1 and IS-2 were 80.54% and 83.60% with RSD values of 2.11% and 5.19%, respectively. These results demonstrated that the recoveries obtained were consistent and reproducible, and the ionization competition (enhancement or suppression effects) between the analytes and the endogenous co-elution was negligible.

The mean stability results (Additional file 1: Table S5) indicated the RE and RSD values of sixteen circulating alkaloids and saponins at three QC levels were in the range from − 14.53% to 14.40% and 13.87%, respectively, indicating that all the analytes were stable under different storage conditions.

In addition, a total of twenty circulating compounds were detected in rat urine. The regression equations, linear ranges and LLOQs for the determination of the analytes in rat urine was shown in Additional file 1: Table S6. Over the considered calibration range, the regression coefficient (*r*^2^) was > 0.9906 for the curves of each analyte. Furthermore, twenty-one circulating compounds were obtained in rat bile, and the calibration curves of twenty-one analytes exhibited the good linearity with *r*^2^ values over 0.9901 (Additional file 1: Table S7) at a certain concentration range in rat bile. From the data, we can see the developed method showed good linearity and sensibility in rat urine and bile.

### Plasma pharmacokinetics of circulating alkaloids and saponins in rats

**Fig. 1:**
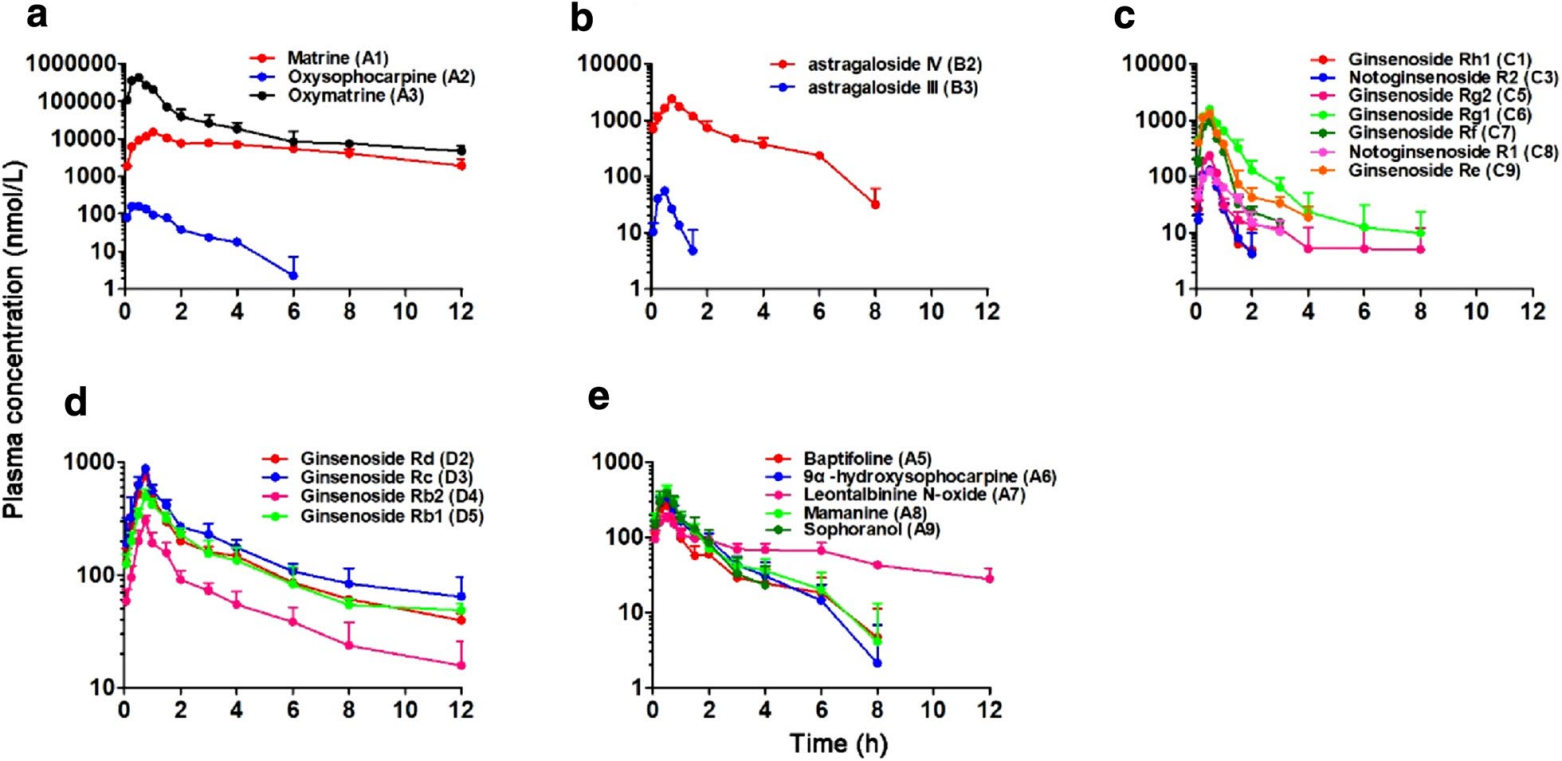
Mean plasma pharmacokinetics of three abundant alkaloids (**a**), two astragalosides (**b**), seven *PPT*-*type* ginsenosides (**c**), four *PPD*-*type* ginsenosides (**d**) and other five minor alkaloids (**e**) over the time after receiving an intravenous 30-min infusion of Kang-Ai injection in rats (6 mL/kg). See Additional file 1: Table S1 for the compounds’ ID and names

Table 1 summarized the PK parameters of circulating alkaloids (**A1–A3**), astragalosides (**B2**, **B3**) and ginsenosides (**C1**, **C3**, **C5–C9**, **D2–D5**) from intravenously dosed Kang-Ai injection. Figure 1a displayed the total plasma concentrations of the alkaloids over time, during and after a 30-min intravenous infusion of Kang-Ai injection in rats. Clearly, the plasma *C*_max_ value of matrine (**A1**) were determined after stopping the infusion for 30 min. After the 30-min infusion, its plasma concentration increased with mean *t*_1/2_ values of 4.33 h. In addition, the *C*_max_ values of oxysophocarpine (**A2**) and oxymatrine (**A3**) were both measured just before stopping the infusion, following the declined plasma concentration with shorter *t*_1/2_ values of 1.78 and 2.73 h, respectively. The mean *V*_SS_ values of **A1**, **A2** and **A3** were 9.85, 25.20 and 220.11 mL/kg, respectively, suggesting these three alkaloids all had small *V*_d_ values. Besides, the CL_tot, p_ values of **A3** was 111.34 mL/h/kg and those of **A1** and **A2** were 1.58 and 12.31, respectively, indicating that **A3** and **A1**, **A2** had moderate and low total clearance, respectively. Furthermore, these circulating alkaloids (**A1–A3**) were not extensively bound with *f*_u-plasma_ values over 90%.

**Table 1:** Pharmacokinetic parameters of circulating alkaloids and saponins in rats after receiving a 30-min infusion of Kang-Ai injection at 6 mL/kg

ID	*t*_1/2_	*C*_max_	AUC_0–12h_	CL_tot, p_	V_SS_	*f*_e-B,0–12h_	CL_B_	*f*_e-U,0–12h_	CL_R_	*f*_u-plasma_
	h	nmol/L	nmol/L*h	mL/h	mL	(%)	mL/h	(%)	mL/h	(%)
A1^#^	4.33 ± 2.18	15.06 ± 3.58	68.61 ± 9.85	1.58 ± 0.36	9.85 ± 2.47	36.55 ± 4.24	0.57 ± 0.09	57.40 ± 4.88	0.90 ± 0.17	92.67 ± 6.81
A2	1.78 ± 1.55	169.87 ± 27.36	257.67 ± 30.97	12.31 ± 1.62	25.20 ± 14.82	51.92 ± 8.98	6.34 ± 1.21	38.60 ± 11.22	4.75 ± 1.53	91.15 ± 4.95
A3^#^	2.73 ± 1.16	422.70 ± 55.50	502.71 ± 93.02	111.34 ± 18.49	220.11 ± 53.82	28.96 ± 3.69	31.95 ± 4.76	63.83 ± 2.28	70.85 ± 10.40	93.24 ± 6.52
B1	–	–	–	–	–	30.40 ± 3.79	–	–	–	–
B2	1.64 ± 0.26	2363.99 ± 251.85	4523.65 ± 464.66	28.39 ± 2.85	64.88 ± 10.23	8.79 ± 1.29	2.49 ± 0.35	17.12 ± 3.13	4.84 ± 0.84	12.35 ± 1.02
B3	0.33 ± 0.13	54.75 ± 10.23	34.29 ± 5.22	206.96 ± 36.55	91.99 ± 21.98	32.61 ± 3.00	67.06 ± 10.52	2.60 ± 0.69	5.25 ± 1.10	11.04 ± 0.86
B4	–	–	–	–	–	–	–	63.43 ± 11.75	–	–
B5	–	–	–	–	–	–	–	51.36 ± 7.44	–	–
C1	0.44 ± 0.39	140.43 ± 20.40	91.16 ± 4.77	198.38 ± 16.38	93.62 ± 45.64	9.71 ± 0.98	19.27 ± 2.44	9.59 ± 1.68	18.97 ± 3.34	46.32 ± 5.03
C2	–	–	–	–	–	24.20 ± 4.93	–	23.37 ± 7.94	–	–
C3	0.29 ± 0.12	128.03 ± 13.27	85.57 ± 10.54	255.80 ± 34.97	99.30 ± 20.42	23.55 ± 1.97	59.94 ± 6.71	10.27 ± 2.49	25.62 ± 3.48	35.24 ± 2.85
C4	–	–	–	–	–	67.93 ± 8.80	–	24.46 ± 3.37	–	–
C5	9.77 ± 13.75	230.90 ± 17.45	192.29 ± 42.13	157.78 ± 74.80	791.61 ± 713.90	20.76 ± 3.37	30.75 ± 17.04	2.23 ± 0.61	3.31 ± 1.86	26.54 ± 3.28
C6	1.20 ± 1.23	1565.45 ± 174.98	1517.34 ± 263.46	156.65 ± 30.66	144.15 ± 64.23	20.71 ± 3.62	33.27 ± 12.03	39.79 ± 5.88	62.25 ± 14.38	86.35 ± 7.32
C7	0.63 ± 0.31	943.15 ± 171.83	674.18 ± 89.61	109.50 ± 15.38	50.05 ± 7.85	13.12 ± 1.68	14.48 ± 3.46	17.77 ± 3.90	19.33 ± 4.45	60.23 ± 7.01
C8	0.75 ± 0.16	121.07 ± 21.08	133.10 ± 17.03	72.70 ± 6.63	71.71 ± 14.31	12.00 ± 1.57	8.73 ± 1.49	72.12 ± 13.01	51.84 ± 5.77	90.36 ± 4.22
C9	1.07 ± 0.84	1338.77 ± 88.07	1000.55 ± 62.12	156.32 ± 11.64	100.55 ± 26.96	28.27 ± 4.16	44.03 ± 5.77	29.54 ± 6.03	46.03 ± 9.04	87.36 ± 6.59
D1	–	–	–	–	–	7.46 ± 2.11	–	–	–	–
D2	5.84 ± 3.13	766.17 ± 57.86	1696.75 ± 124.20	5.83 ± 0.67	35.69 ± 11.39	5.55 ± 1.02	0.33 ± 0.08	19.00 ± 5.33	1.11 ± 0.32	0.75 ± 0.08
D3	6.29 ± 3.17	877.14 ± 39.49	2165.94 ± 186.38	5.60 ± 1.41	37.81 ± 10.15	–	–	10.48 ± 1.31	0.58 ± 0.15	1.12 ± 0.12
D4	4.12 ± 0.83	301.33 ± 34.23	698.17 ± 105.74	7.80 ± 1.14	39.58 ± 7.30	–	–	27.80 ± 4.47	2.14 ± 0.27	0.67 ± 0.05
D5	7.22 ± 7.03	497.21 ± 75.11	1536.70 ± 223.18	6.16 ± 1.57	46.96 ± 19.87	–	–	6.41 ± 1.47	0.38 ± 0.09	0.96 ± 0.09
E1	–	–	–	–	–	27.80 ± 7.01	–	–	–	–
E2	–	–	–	–	–	46.95 ± 6.46	–	–	–	–
E3	–	–	–	–	–	31.31 ± 6.25	–	–	–	–
E4	–	–	–	–	–	29.36 ± 3.14	–	–	–	–
A4	–	–	–	–	–	11.14 ± 1.05	–	42.80 ± 14.69	–	–
A5	2.15 ± 0.50	264.42 ± 47.19	380.06 ± 117.01	26.77 ± 11.13	55.47 ± 6.92	10.30 ± 1.11	2.68 ± 0.82	37.56 ± 15.13	10.99 ± 8.12	–
A6	2.16 ± 1.28	344.22 ± 40.74	509.97 ± 93.65	47.70 ± 10.72	93.63 ± 24.86	12.50 ± 1.32	5.86 ± 1.10	12.92 ± 4.98	6.23 ± 2.97	–
A7	5.73 ± 1.74	202.37 ± 19.05	777.15 ± 81.47	20.03 ± 4.84	150.70 ± 19.02	7.30 ± 1.41	1.42 ± 0.17	61.05 ± 10.61	12.32 ± 4.14	–
A8	2.32 ± 1.03	421.27 ± 68.11	567.07 ± 114.95	7.93 ± 1.77	16.89 ± 5.16	34.12 ± 4.23	2.67 ± 0.46	15.28 ± 5.11	1.23 ± 0.50	–
A9	0.82 ± 0.37	396.86 ± 49.71	486.66 ± 132.17	56.05 ± 22.70	53.50 ± 7.71	1.27 ± 0.20	0.68 ± 0.16	59.77 ± 8.80	34.24 ± 17.17	–
B6	–	–	–	–	–	–	–	74.96 ± 6.01	–	–
C10	–	–	–	–	–	–	–	40.28 ± 5.48	–	–
C11	–	–	–	–	–	–	–	48.30 ± 10.27	–	–
C12	–	–	–	–	–	–	–	36.16 ± 7.04	–	–
C13	–	–	–	–	–	–	–	38.72 ± 6.57	–	–

^#^ Means the units of C_max_ and AUC_0–12h_ for matrine and oxymatrine are μmol/L and μmol/L*h, respectively. See Additional file 1: Table S1 for the compounds’ ID and names

The plasma concentration–time curves and PK parameters of astragaloside IV (**B2**) and III (**B3**) were shown in Fig. 1b and Table 1, respectively. The plasma *C*_max_ (2363.99 nmol/L) and AUC_0-∞_ (4523.65 nmol/L*h) values of **B2** were far higher than those of **B3** with *C*_max_ and AUC_0-∞_ values of (54.75 nmol/L) and AUC_0-∞_ (34.29 nmol/L*h), due to the differences in the compounds’ doses from the injection. Besides, the *t*_1/2_ values of these two astragalosides were both small and less than 2 h. Similarly, **B2** and **B3** both exhibited the small *V*_d_ values (< 100 mL/kg), whereas **B2** (28.39 mL/h/kg) and **B3** (206.96 mL/h/kg) had low and moderate total clearance, respectively. Protein binding assay demonstrated that **B2** and **B3** were extensively bound in rat plasma (nearly 90%).

These circulating ginsenosides in plasma could be classified into two categories, protopanaxatriol (*PPT*) type ginsenosides (**C1**, **C3**, **C5–C9**) and protopanaxadiol (*PPD*) type ginsenosides (**D2–D5**). Fig. 1c, d summarized the plasma concentrations of the *PPT*-*type* ginsenosides and *PPD*-*type* ginsenosides over time, respectively. It was notably shown that the *T*_max_ values of *PPT*-*type* ginsenosides were 30 min, whereas those of *PPD*-*type* ginsenosides were measured after stopping infusion for 15 min. In addition, the *C*_4h_ values of the *PPT*-*type* ginsenosides were very low, while those of the *PPD*-*type* ginsenosides still maintained a moderate plasma concentration. These suggested that the *PPT*-*type* ginsenosides possessed the longer MRT, and were eliminated more quickly than *PPD*-*type* ginsenosides. Another strong evidence was that CL_tot, p_ values of *PPT*-*type* ginsenosides (72.70–255.80 mL/h/kg) were approximately 10 times of those of *PPT*-*type* ginsenosides (5.60–7.80 mL/h/kg). Besides, except for ginsenoside Rg2 (**C5**) about the *t*_1/2_ value of 9.77 h, the *PPT*-*type* ginsenosides exhibited the shorter elimination half-lives values of i.e., 0.29–1.20 h for ginsenoside Rh1 (**C1**), Rg1 (**C6**), Rf (**C7**), Re (**C9**) and notoginsenoside R1 (**C8**), R2 (**C3**) than those of 4.12–7.22 h for ginsenosides Rd (**D2**), Rc (**D3**), Rb2 (**D4**) and Rb1 (**D5**). The *V*_SS_ values of *PPT*-*type* ginsenosides and *PPD*-*type* ginsenosides were 50.05–791.61 and 35.69–46.96 mL/kg, respectively, indicating that the *PPT*-*type* ginsenosides were more easily distributed in various body fluids and tissues than the *PPD*-*type* ginsenosides. Additionally, the *PPT*-*type* ginsenosides (**C1**, **C3**, **C5–C9**) were not significantly bound, whereas the *PPD*-*type* ginsenosides (**D2–D5**) were obviously bound (around 99%) in rat plasma.

Moreover, due to lack of the available authentic standards, virtual quantification of other main circulating herbal compounds in rat plasma, including baptifoline (**A5**), 9α-hydroxy-sophocarpine (**A6**), leontalbinine *N*-oxide (**A7**), mamanine (**A8**), sophoranol (**A9**), were achieved using the calibration curve of an available reference standard of an analog that bore close structural similarity to the analyte. Figure 1e, Table 1 showed their mean plasma concentration–time curves and calculated relative PK parameters, respectively. The *C*_max_ values ranged from 202.37 to 421.27 nmol/L, and the *T*_max_ values were all determined just before stopping the infusion. Besides, **A7** exhibited the longest MRT (7.94 h) and *t*_1/2_ (5.73 h) values among these five alkaloids. The small *V*_SS_ (16.89–150.70 mL/kg) and CL_tot, p_ (7.93–56.05 mL/h/kg) values indicated that these alkaloids were slowly distributed in various body fluids and tissues, and slowly cleared from the systemic circulation.

**Fig. 2:**
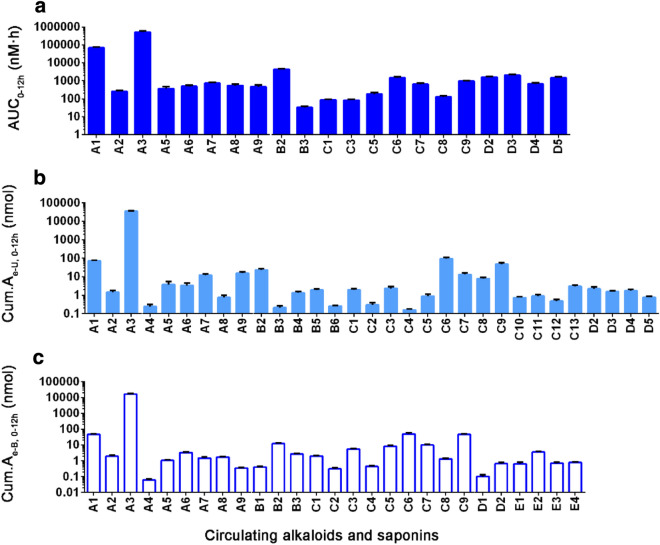
Systemic exposure in plasma (**a**), renal excretion in urine (**b**) and hepatobiliary excretion in bile (**c**) of circulating alkaloids, astragalosides and ginsenosides after a 30-min intravenous infusion of Kang-Ai injection to rats (6 mL/kg). See Additional file 1: Table S1 for the compounds’ ID and names

To better demonstrate the systemic exposure in rat plasma, the comparison of the AUC_0–12h_ values of circulation alkaloids and saponins were displayed in Fig. 2a. Obviously, oxymatrine (**A3**) exhibited the most abundant exposure with 502.71 μmol/L*h, due to the most abundant dose level from Kang-Ai injection. Additionally, **A1**, **B2**, **D3**, **D2**, **D5**, **C6**, **C9**, **A7**, **D4**, **C7**, **A8**, **A6**, **A9**, **A5**, **A2**, **C5**, **C8**, in descending order, exhibited substantially higher total levels of plasma exposure with the AUC_0–12h_ values over 100 nmol/L*h, whereas the levels of plasma exposure to other compounds (**B3**, **C1**, **C3**) were quite low, due to their low compound dose from Kang-Ai injection.

### Urinary and biliary excretion of circulating alkaloids and saponins in rats

Table 1 summarized the urinary and biliary excretion parameters of circulating alkaloids and saponins. Meanwhile, Fig. 2b, c showed their cumulative excretion amounts in urine and bile, respectively. For the circulating alkaloids (**A1–A3**), the renal and hepatobiliary excretion of the unchanged compounds represented the major routes of elimination (Table 1). This was attributed that **A1** (57.40%), **A2** (38.60%) and **A3** (63.83%) exhibited an extraordinarily high *f*_e-U,0–12h_ values, the sum of which with the *f*_e-B,0–12h_ values (36.55%, 51.92%, 28.96%, respectively) exceeded 90% of individual dose levels.

Astragaloside III (**B3**) was eliminated more extensively via hepatobiliary excretion (*f*_e-B_, 67.06%) than renal excretion (*f*_e-U_, 5.25%) (Table 1). Unlike for **B3**, renal excretion was a minor elimination pathway for astragaloside IV (**B2**) (*f*_e-U_, 4.84%), and the same fact was that the fraction of dosed **B2** was excreted into bile (*f*_e-B_) about 2.49%.

The *PPT*-*type* ginsenosides (**C1–C9**), except ginsenoside Rg2 (**C5**), all exhibited the large *f*_e-U_ values (9.59–72.17%) (Table 1), suggesting that the renal excretion was the predominant elimination route responsible for their systemic clearance. Similarly, the *PPD*-*type* ginsenosides (**D2–D5**), exhibited moderate *f*_e-U_ values (6.41–27.8%), which was around a half of the values of *PPT*-*type* ginsenosides. In addition, in rat bile, the *PPT*-*type* ginsenosides were the most abundant herbal compounds with *f*_e-B_ values of 9.71–67.93%, whereas only two *PPD*-*type* ginsenosides, ginsenoside F2 (**D1**) and Rd (**D2**) could be detected and displayed the small *f*_e-B_ values of 7.46% and 5.55%, respectively. Consistent with the inter-compound differences in CL_tot, p_ values, the CL_R_ and CL_B_ values for these *PPT*-*type* ginsenosides (**C1–C9**) were significantly greater than those respect values for the *PPD*-*type* ginsenosides (**D1–D5**).

Moreover, other alkaloids (**A4–A9**) were eliminated more extensively unchanged via the renal excretion (*f*_e-U_, 12.92–61.05%) than the hepatobiliary excretion (*f*_e-B_, 1.27–34.12%) (Table 1). The CL_R_ and CL_B_ values for the alkaloids (**A4–A9**) kept in line with their CL_tot, p_ values. Another astragaloside, astragaloside VII (**B6**) was excreted 74.96% of the dose derived from Kang-Ai injection to urine. Another four *PPT*-*type* ginsenosides, ginsenosides R1 (**C10**), Re4 (**C11**), notoginsenoside R3 (**C12**) and 20-gluco-ginsenoside Rf (**C13**) were all subjected to be eliminated by the renal excretion with *f*_e-U_ values of 36.16–48.30%, whereas their hepatobiliary excretion appeared to be negligible.

### Inhibitory effects of Kang-Ai injection and herbal compounds towards each CYP and UGT isozyme

**Fig. 3:**
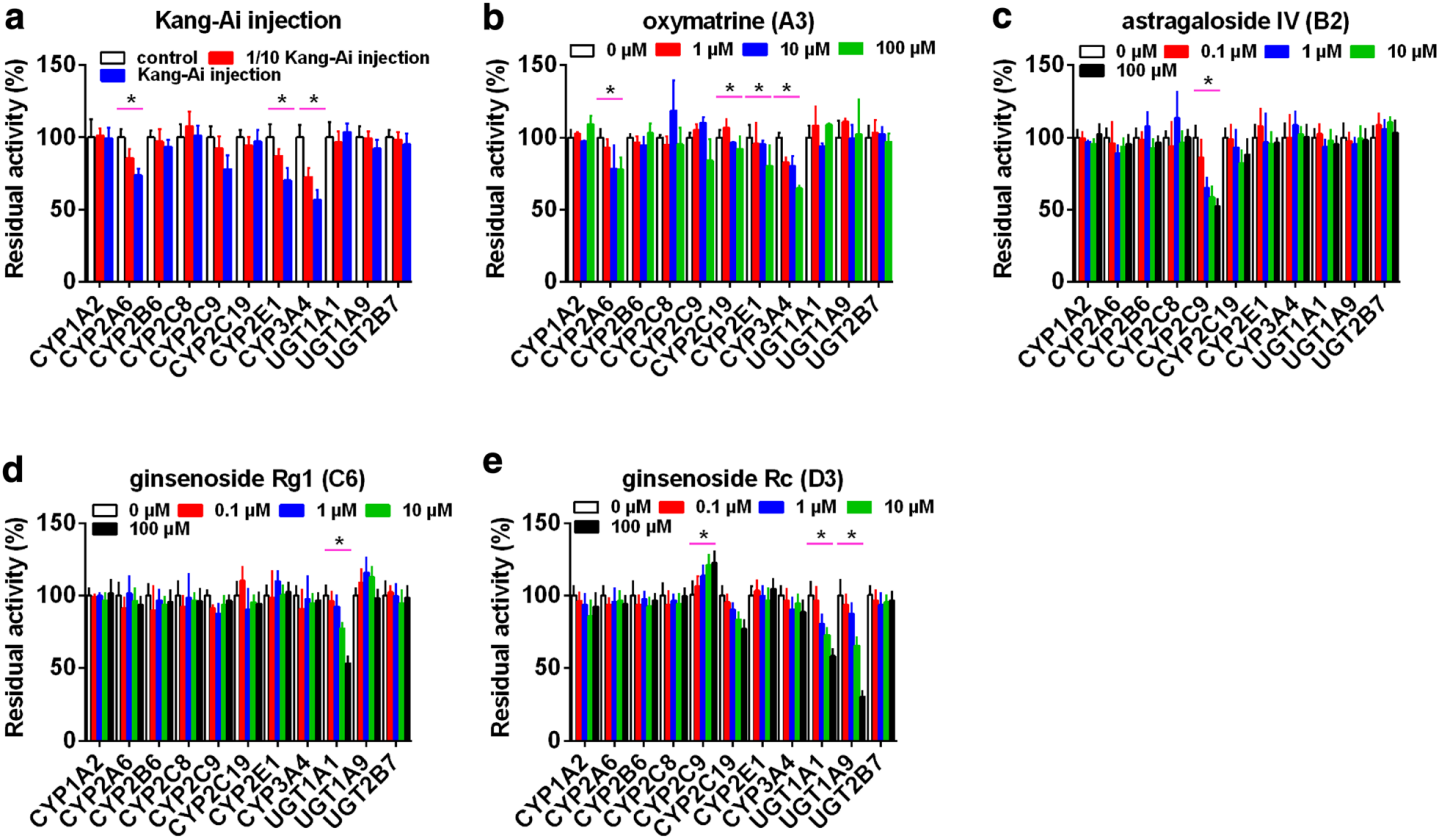
The effects of several circulating compounds towards eight expressed CYP isozymes and three recombinant UGT enzymes. **a** Kang-Ai injection, **b** oxymatrine, **c** astragaloside IV, **d** ginsenosides Rg1, and **e** ginsenoside Rc; The probe substrates were incubated at 37 °C in the absence (control, 0 μM) and presence of tested mixture and compounds (Kang-Ai injection and samples that Kang-Ai injection was diluted 10 times; 1, 10, and 100 μM for oxymatrine; 0.1, 1, 10 and 100 μM for astragalosides and ginsenosides). Data represent the mean ± standard deviation of triplicate, (* compared with those of control, * p < 0.05)

Kang-Ai injection showed inhibitory effects towards CYP2A6, 2E1 and 3A4 with residual activity of 73.63%, 70.28% and 56.92%, respectively (Fig. 3a). Meanwhile, Kang-Ai injection almost exhibited no inhibitory effects against other CYP and UGT enzymes (Fig. 3a). Based on Table 1, matrine (**A1**), oxymatrine (**A3**), astragaloside IV (**B2**), ginsenosides Rg1 (**C6**), Rf (**C7**), Re (**C9**), Rd (**D2**), Rc (**D3**), Rb1 (**D5**) exhibited the C_max_ values about or over 0.5 μmol/L after intravenous Kang-Ai injection. Therefore, these nine major circulating compounds were selected to evaluate the inhibitory effects toward several common CYP and UGT enzymes. **A1** (100 μM) displayed inhibitory effects against CYP3A4 (66.63%) (Additional file 1: Fig. S5A), whereas **A3** (100 μM) could inhibit the metabolic activity of CYP2A6 (77.63%), 2E1 (80.28%), and 3A4 (64.92%) (Fig. 3b). In addition, **B2** (100 μM) exhibited inhibitory activity towards CYP2C9 (52.36%) (Fig. 3c). Besides, *PPT*-*type* ginsenosides, **C6** (100 μM) (Fig. 3d) and **C9** (100 μM) (Additional file 1: Fig. S5C) mainly inhibited the function of UGT1A1 (53.26%) and CYP2C8 (30.25%), respectively, while almost no changes were observed for **C7** (100 μM) against these CYP and UGT enzymes (Additional file 1: Fig. S5B). Furthermore, **D2** (100 μM) exhibited inhibition on the metabolic activity of CYP2B6 (30.36%) and 3A4 (42.36%) (Additional file 1: Fig. S5D), whereas **D3** (100 μM) (Fig. 3e) and **D5** (100 μM) (Additional file 1: Fig. S5E) showed inhibitory effects on UGT1A9 (30.26%) and CYP2C9 (15.36%), respectively.

**Fig. 4:**
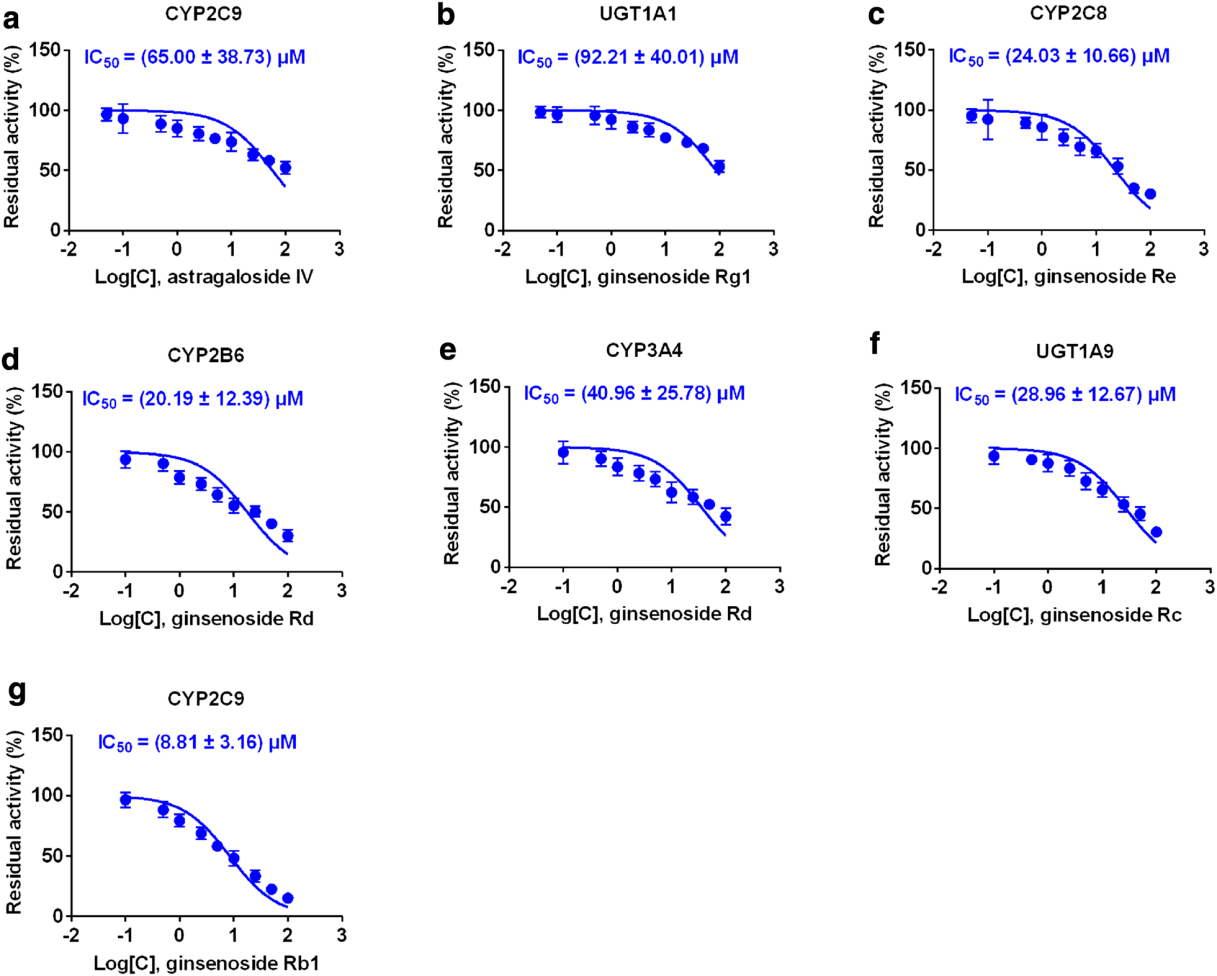
The IC50 values of astragaloside IV against CYP2C9 (**a**), ginsenoside Rg1 against UGT1A1 (**b**), ginsenoside Re towards CYP2C8 (**c**), ginsenoside Rd towards CYP2B6 (**d**) and CYP3A4 (**e**), ginsenoside Rc against UGT1A9 (**f**), and ginsenoside Rb1 against CYP2C9 (**g**). The data were fit to log (concentration) and normalized response equations. Each data point represented the mean value ± the S.D. of triplicate determination

Moreover, the inhibition data (Fig. 4) were fit to log (I) and normalized response equations to obtain the IC_50_ values. The inhibition trend of the abundant compounds towards these CYP and UGT enzymes showed in a dose dependent manner, and their IC_50_ values were 65.00 (**B2** towards CYP2C9), 92.21 (**C6** against UGT1A1), 24.03 (**C9** towards CYP2C8), 20.19 (**D2** against CYP2B6), 40.96 (**D2** towards CYP3A4), 28.96 (**D3** against UGT1A9) and 8.81 μM (**D5** towards CYP2C9), respectively (Additional file 1: Table S8). The inhibition mechanism was further evaluated based on three inhibition kinetic models.

### Analysis of inhibition mechanism

The AIC and SC values (Table 2) were obtained after the inhibition data were modeled by three conventional inhibition equation (i.e., competitive, noncompetitive and mixed-type). Based on the smallest AIC and SC values principle, **C6** displayed competitive inhibition against UGT1A1, while **C9** exhibited mixed-type inhibition against CYP2C8. Noncompetitive inhibition kinetics were observed for **B2** against CYP2C9, **D2** towards CYP2B6 and 3A4, **D3** against UGT1A9 and **D5** towards CYP2C9.

**Table 2:** Inhibition parameters of circulating alkaloids and saponins against several CYP and UGT isozymes

Compounds	Isozymes	Inhibition type	K_i_ (μM)	α	*R*^2^	AICs	SCs	Selection	C_max_/K_i_
Astragaloside IV (B2)	CYP2C9	Competitive	17.4 ± 3.7	–	0.9480	− 113.3	− 110.3		
		Noncompetitive	55.8 ± 5.3	–	0.9708	− 124.8	− 121.8	√	0.04
		Mixed-type	45.0 ± 16.6	1.5 ± 1.0	0.9713	− 123.1	− 119.2		
Ginsenoside Rg1 (C6)	UGT1A1	Competitive	140.6 ± 10.5	–	0.9952	176.4	179.4	√	0.01
		Noncompetitive	187.2 ± 12.7	–	0.9943	179.8	182.8		
		Mixed-type	147.3 ± 29.9	8.3 ± 37.2	0.9952	178.7	182.7		
Ginsenoside Re (C9)	CYP2C8	Competitive	8.2 ± 1.1	–	0.9790	− 121.3	− 118.3		
		Noncompetitive	26.5 ± 2.2	–	0.9793	− 121.6	− 118.6		
		Mixed-type	13.3 ± 2.9	4.2 ± 2.3	0.9862	− 127.8	− 123.8	√	0.1
Ginsenoside Rd (D2)	CYP2B6	Competitive	10.2 ± 2.1	–	0.9484	19.4	22.4		
		Noncompetitive	37.2 ± 2.3	–	0.9877	− 9.2	− 6.2	√	0.02
		Mixed-type	36.3 ± 10.2	1.0 ± 0.4	0.9877	− 7.2	− 3.2		
Ginsenoside Rd (D2)	CYP3A4	Competitive	8.7 ± 2.3	–	0.9226	− 31.9	− 28.9		
		Noncompetitive	32.8 ± 2.2	–	0.9846	− 64.2	− 61.2	√	0.02
		Mixed-type	44.2 ± 16.2	0.6 ± 0.3	0.9854	− 63.3	− 59.3		
Ginsenoside Rc (D3)	UGT1A9	Competitive	8.9 ± 1.5	–	0.9634	122.8	125.8		
		Noncompetitive	22.3 ± 1.4	–	0.9877	100.9	103.9	√	0.04
		Mixed-type	27.0 ± 7.5	0.7 ± 0.3	0.9881	102.3	106.3		
Ginsenoside Rb1 (D5)	CYP2C9	Competitive	8.2 ± 1.0	–	0.9812	− 115.9	− 112.9		
		Noncompetitive	16.1 ± 0.9	–	0.9921	− 133.2	− 130.3	√	0.03
		Mixed-type	18.8 ± 4.3	0.7 ± 0.3	0.9923	− 131.8	− 127.9		

Data represent the mean ± standard deviation of triplicate

C_max_/K_i_ also named [I]/K_i_ standard ([I]/Ki < 0.1, low possibility DDI; 1 > [I]/Ki > 0.1, medium possibility DDI; [I]/Ki > 1, high possibility DDI)

**Fig. 5:**
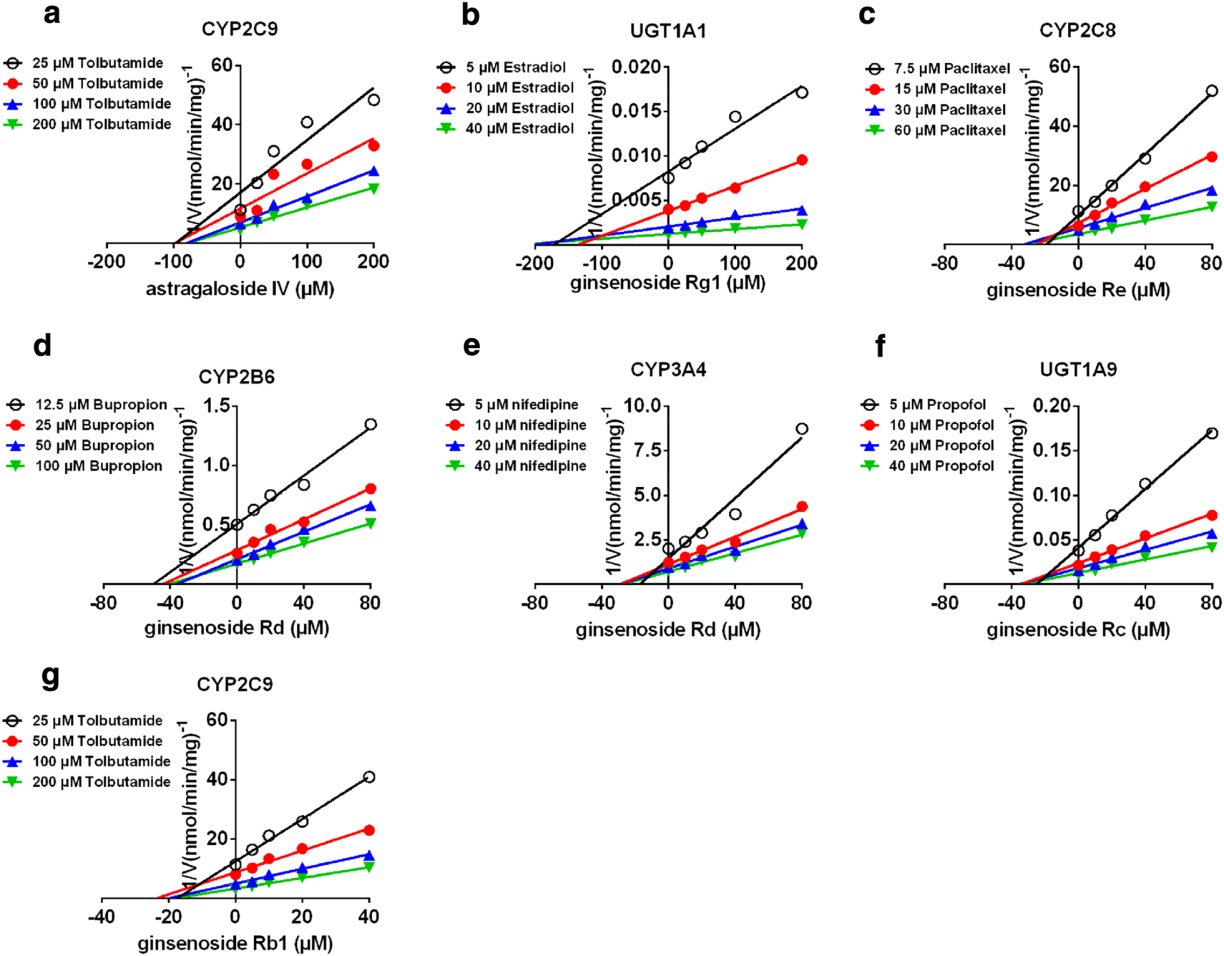
The Dixon plots for the inhibition effects of circulating saponins against recombinant CYP and UGT isozymes. **a** Inhibition effect of astragaloside IV against tolbutamide-4-hydroxylation for CYP2C9; **b** Inhibition effect of ginsenoside Rg1 towards β-estradiol-3-*O*-glucuronidation for UGT1A1; **c** Inhibition effect of ginsenoside Re against paclitaxel-6-hydroxylation for CYP2C8; **d** Inhibition effect of ginsenoside Rd towards bupropion-hydroxylation for CYP2B6; **e** Inhibition effect of ginsenoside Rd against nifedipine-oxidation for CYP3A4; **f** Inhibition effect of ginsenoside Rc towards propofol-*O*-glucuronidation for UGT1A9; **g** Inhibition effect of ginsenoside Rb1 against tolbutamide-4-hydroxylation for CYP2C9; All data represent the mean ± SD of triplicate determinations

Furthermore, the Dixon plots for **B2** against CYP2C9 (Fig. 5a), **C6** towards UGT1A1 (Fig. 5b), **C9** against CYP2C8 (Fig. 5c), **D2** towards CYP2B6 (Fig. 5d) and 3A4 (Fig. 5e), **D3** against UGT1A9 (Fig. 5f) and **D5** towards CYP2C9 (Fig. 5g) also provided strong evidences to support this judgment about the inhibition mechanism. Their respective K_i_ values were 55.8, 140.6, 13.3, 37.2, 32.8, 22.3 and 16.1 μM (Table 2). These findings suggested that the major circulating compounds have weak or no inhibition on the metabolic activity of common CYP and UGT enzymes with K_i_ values all over 10 μM.

### Potential DDI risks

Based on the [I]/K_i_ values in vitro, potential DDI risks in vivo could be predicted [27, 28]. Traditionally, [I]/Ki < 0.1, low possibility DDI; 1 > [I]/Ki > 0.1, medium possibility DDI; [I]/Ki > 1, high possibility DDI [18]. In this study, [I]/K_i_ values for these herbal compounds were all no more than 0.1 (Table 2). Therefore, it seemed impossible to trigger DDI when Kang-Ai injection were co-administrated with clinical drugs which were metabolized by these enzymes.

## Discussion

Bioactive compounds with adequate abundance in herbal injection and favorable PK properties are mostly believed to be responsible for the pharmacological effects and therapeutic efficacy [4]. In the current study, alkaloids and saponins derived from Kang-Ai injection are two classes of bioactive compounds which are likely responsible for the adjuvant therapy of many cancers (Additional file 1: Table S1) [6, 8]. After dosing, oxymatrine (**A3**), matrine (**A1**), astragaloside IV (**B2**), ginsenosides Rc (**D3**), Rd (**D2**), Rb1 (**D5**), Rg1 (**C6**) and Re (**C9**), all exhibited considerably high levels of systemic exposure (over 1000 nmol/L*h) (Fig. 2a), indicating that these circulating compounds are worth special consideration in the pharmacological research. Besides, the abundant exposure of **A1** may be attributed the metabolism of **A3** by CYP3As [29]. In addition, the considerable systemic exposure for **A3**, **A1**, **D3**, **D2** and **D5**, seemed to be correlated with the significantly longer *t*_1/2_ values of 2.73–7.22 h than those of **B2**, **C6** and **C9** (1.07–1.64 h) (Table 1). This phenomenon that long elimination half-life could significantly counterbalance influence on systemic exposure to herbal compounds with poor bioavailability has been proved previously [30].

Except the systemic exposure of major circulating compounds, we also investigate their elimination in the current study (Table 1). After dosing Kang-Ai injection, oxymatrine (**A3**) and matrine (**A1**) mainly involved in the renal and hepatobiliary excretion of the parent compounds (over 90%), of which parts of circulated **A1** was originated from the metabolism of **A3** by CYP3A4 [11]. Only about 25% of dosed astragaloside IV (**B2**) were detected as parent compound in urine and bile, and this was probably attributed to the de-glycosylation of **B2** to its sapogenin [15]. Additionally, ginsenosides Rg1 (**C6**) and Re (**C9**) were eliminated mainly to urine and bile (nearly 60%), whereas it proved that approximately 25% of ginsenoside Rd (**D2**) was excreted via renal and hepatobiliary elimination. Ginsenosides Rc (**D3**) and Rb1 (**D5**) were not detected in bile, and mainly were eliminated through renal excretion. Except the in vivo metabolism, i.e., oxidation and de-glycosylation [16], the delivery to the rat tissues of circulating herbal compounds through blood flow were also an important factor to determine the systemic exposure and excretion of circulating compounds [31]. The V_SS_ values of dosed **A3**, **C6** and **C9** were 100.55–220.11 mL/kg, which were near the rat extracellular volume (300 mL/kg) [32], indicating that these herbal compounds could be distributed evenly in various body tissues, except several barriered tissues. However, this elimination route warrant future investigation.

In addition, the obtained PK parameters (Table 1) also could enable us to compare the plasma concentrations of the alkaloids and saponins with the reported effective concentrations (IC_50_ or EC_50_ values) for pharmacological activities. Oxymatrine (**A3**) exhibited the most abundant concentration (422.70 μmol/L) after dosing Kang-Ai injection, which is still obviously below the reported effective concentrations (473.48–3787.84 μmol/L) for several tumor cell lines, including HepG2, MCF-7, HeLa, A549 cells [33]. However, for SW-620, BGC823, A375 cells, **A3** is sufficiently effective to inhibit the tumor cell viability (0.10–40.00 μmol/L) [33]. Similar results were obtained for matrine (**A1**) and its derivatives [34], astragaloside IV (**B2**) [35], and several main ginsenosides [36]. Although the low plasma concentrations do not result in the ineffectiveness of Kang-Ai injection for the therapeutic effects, they may limit the contribution of bioactive compounds to the current adjuvant therapy. How to achieve the higher plasma concentrations is an important idea to develop new and superior adjuvant agents derived from the current Kang-Ai injection.

Kang-Ai injection are the combinations of two herbs and Kushensu, of which complex chemical compositions increased the DDI risks. Usually, the induction and inhibition of CYP and UGT enzymes can facilitate the metabolism and elimination of their drug substrates and caused unexpected adverse effect. Previously, oxymatrine (**A3**) was subjected to be metabolized to matrine (**A1**) rapidly in pooled HLM (*K*_m_ = 220.78 μM) and HIM (*K*_m_ = 150.22 μM), and CYP3A4 (*K*_m_ = 341.35 μM) isozyme greatly contributed to this transformation [37]. However, **A1** could not be extensively metabolized by CYPs and UGTs [38]. In addition, considering that most of clinical drugs or endogenous compounds are metabolized or eliminated by CYP1A2 (9%), 2A6, 2B6 (2%), 2C8, 2C9 (16%), 2C19 (12%), 2E1 (2%), 3A4 (46%), and UGT1A1 (15%), 1A9, 2B7 (35%) [39, 40], we focused on the inhibitory effects of herbal compounds derived from Kang-Ai injection against these enzymes above.

Our results showed that Kang-Ai injection demonstrated weak inhibitory effects against CYP2A6, 2E1 and 3A4 (Fig. 3a), which could be mainly attributed to the contribution of oxymatrine. This is because the content level of oxymatrine in Kang-Ai injection (37731.41 μM) is far higher than those of other herbal compounds (0.23–158.95 μM) (Additional file 1: Table S1) [9]. Furthermore, it was notable that the de-glycosylation of astragaloside IV (**B2**) to sapogenin-cycloastragenol strongly increased the inhibitory effects towards UGT1A8 (*K*_i_ = 0.034 µM) and 2B7 (*K*_i_ = 20.98 µM) [15]. Interestingly, naturally occurring alkaloids and ginsenosides, including **A1**, **A3,** ginsenosides Rg1 (**C6**), Rf (**C7**), Re (**C9**), Rd (**D2**), Rc (**D3**) and Rb1 (**D5**), were unlikely to cause clinically relevant DDI mediated via the induction or inhibition of most CYPs or UGTs involving in drug metabolism in vivo, which agreed with previous study [41].

Identification of the induction or inhibition of the abundant circulating alkaloids and saponins towards uptake and efflux transporters was another significant risk factor for the DDI. In clinics, oxymatrine (**A3**) could interfere with hOCT1-mediated hepatic uptake and renal elimination, and intestinal absorption by hOCT3, whereas matrine (**A1**) only had the potential to decrease hOCT3 expressed in enterocytes [42]. Besides, **A1** and **A3** did not demonstrate the inhibition of P-gp in vitro and in vivo [43]. Additionally, it was reported that astragaloside IV (**B2**) could induce the protein levels of P-gp, MRP1, MRP3, but produced the inhibitory effects on BCRP [44]. In contrast, in MDR1-MDCKII cells, **B2** only exhibited weak inhibition activity of 8.22% when treated with 100 μM [43]. Recently, several scholars got the fact that **B2** could induce the upregulation of P-gp and BCRP, and might be a potential substrate of P-gp [45]. For ginsenosides, ginsenoside Rg1 (**C6**) was potential inhibitor of NTCP (IC_50_ = 50.49 μM), whereas ginsenoside Re (**C9**) was potential substrates of NTCP [46]. Further, DDI between ginsenosides Rb1 (**D5**), Rc (**D3**), Rd (**D2**) and methotrexate (a probe substrate for Mrp2) might occur owing to the decrease in the mRNA and protein levels of Mrp2 [47]. In addition, **D5**, **D3**, **D2** and **D9** were predicted to possess the high potential for human OATP1B3-mediated drug interactions [16]. Moreover, *PPT* and *PPD*, the aglycones of *PPT*-*type* ginsenosides and *PPD*-*type* ginsenosides, respectively, were potent P-gp inhibitors, suggesting the unpredictable interactions when ginsenosides were co-administered with the P-gp substrate drugs [48]. The interactions of complex circulating compounds and clinical drugs based on transporters may be one of the important compatibility mechanisms for Kang-Ai injection.

In clinical consumption, the adverse drug reactions (ADR) derived from herbal injections, should be gaining more attention. It was essential to evaluate the objectivity of ADR, develop a reasonable application strategy, and ensure the security and validity of Kang-Ai injection. Data and information obtained from PK studies on the circulating alkaloids and saponins could help understand the complex action of herbal injections, and predict a variety of events related to the efficacy and toxicity of herbal injections. In Kang-Ai injection, the most abundant compounds, oxymatrine (**A3**) and matrine (**A1**) both could cause the hepatic toxicities, of which the toxicity of **A1** was greater than that of **A3** [49]. The elevated metabolism of **A3** by CYP3A4 may result in more severe liver damage. Therefore, the clarification of the PK studies could be helpful in deciphering the ADR and hepatorenal toxicity of herbal injections.

## Conclusion

Herein, nine alkaloids, six astragalosides and twenty-two ginsenosides were the main circulating compounds after dosing Kang-Ai injection. Oxymatrine (**A3**), matrine (**A1**), astragaloside IV (**B2**), ginsenosides Rc (**D3**), Rd (**D2**), Rb1 (**D5**), Rg1 (**C6**), Re (**C9**), Rf (**C7**), and Rb2 (**D4**) exhibited high exposure in plasma. Renal and hepatobiliary excretion were their major elimination pathways. In addition, *PPD*-*type* ginsenosides (**D2–D5**) were extensively bound with *f*_u-plasma_ values approximately 1%, whereas alkaloids and *PPT*-*type* ginsenosides (**C6**, **C7**, **C9**) displayed higher *f*_u-plasma_ values (60.23–93.24%). Furthermore, **A1**, **A3**, **B2**, **C6**, **C7**, **C9**, **D2**, **D3** and **D5** displayed weak inhibition or no inhibition towards several common CYP and UGT enzymes with IC_50_ values ranging from 8.81 to 92.21 μM, suggesting negligible DDI risk. Taken together, the current PK and excretion information gained here could serve as a crucial step in understanding the roles of circulating alkaloids, astragalosides and ginsenosides in therapeutic actions of Kang-Ai injection. These results here will also inform the negligible DDI risks in clinical use of Kang-Ai injection.

## Supplementary information

**Additional file 1: Table S1.** Information of unchanged compounds in rats after a 30 min-infusion of Kang-Ai injection (6 mL/kg) by UHPLC/Q-TOF–MS. **Table S2.** Linear correlation parameters, limits of detection (LODs) and lower limits of quantification (LLOQs) of each circulating compound in rat plasma after a 30 min-infusion of Kang-Ai injection (6 mL/kg). **Table S3.** Relative standard deviation (RSD) values of intra-day and inter-day precisions, and of sixteen compounds in rat plasma after a 30 min-infusion of Kang-Ai injection (6 mL/kg). **Table S4.** Matrix effect and extraction recovery of each compound and two internal standard (IS) in rat plasma after a 30 min-infusion of Kang-Ai injection (6 mL/kg). **Table S5.** Stability results of each compound and internal standard in rat plasma after a 30 min-infusion of Kang-Ai injection (6 mL/kg). **Table S6.** Linear correlation parameters, LLOQs of twenty analytes in rat urine after a 30 min-infusion of Kang-Ai injection (6 mL/kg). **Table S7.** Linear correlation parameters, LLOQs of twenty-one analytes in rat bile after a 30 min-infusion of Kang-Ai injection (6 mL/kg). **Table S8.** The IC_50_ values of six circulating alkaloids and saponins towards several recombinant CYP and UGT isozymes. Data represent the mean ± standard deviation of triplicate. **Fig S1.** Effects of different incubation conditions on the metabolism of mephenytoin by human CYP2C19 (mean ± SD, n = 3). (A) Tris buffer concentration; (B) pH values; (C) MgCl_2_ concentration; (D) NADPH concentration; (E) incubation time; (F) protein concentration. **Fig S2.** Effects of different incubation conditions on the metabolism of estradiol by UGT1A1. (A) Tris buffer concentration; (B) pH values; (C) MgCl_2_ concentration; (D) UDPGA concentration; (E) incubation time; (F) protein concentration. Data represent the mean ± standard deviation of triplicate. **Fig S3.** Chemical structures (A) and content levels (B) of circulating alkaloids, astragalosides and ginsenosides in rats after intravenous Kang-Ai injection (6 mL/kg). See Additional file 1: Table S1 for compounds identification and names. **Fig S4.** Representative MRM chromatograms of sixteen circulating compounds in plasma. (A) blank plasma, (B) blank plasma spiked with sixteen analytes at LLOQs, (C) rat plasma samples at 0.5 h after an intravenous 30 min-infusion of Kang-Ai injection. **Fig. S5** Effects of several circulating compounds towards eight expressed CYP isozymes and three recombinant UGT enzymes. (A) matrine, (B) ginsenoside Rf, (C) ginsenoside Re, (D) ginsenoside Rd, (E) ginsenoside Rb1; The probe substrates were incubated at 37 °C in the absence (control, 0 μM) and presence of tested compounds (1, 10, and 100 μM for matrine; 0.1, 1, 10 and 100 μM for astragalosides and ginsenosides). Data represent the mean ± standard deviation of triplicate, (* compared with those of control, * *p *< 0.05).

## Data Availability

Most of the data in the present study are included in this article and its additional information.

## Abbreviations

4-MU: 4-methylumbelliferone
AIC: Akaike information criterion
BCRP: Breast cancer resistance protein
CYP: Cytochrome P450
DDI: Drug–drug interactions
hOCT: Human organic cation transporter
HLM: Human liver microsomes
IS: Internal standard
LLOQ: Lower limit of quantification
MDR1/P-gp: Multidrug resistance protein 1
MRPs: Multidrug resistance-associated proteins
MRM: Multiple reactions monitoring
NADPH: Nicotinamide adenine dinucleotide phosphate
OATP: Organic anion transporting polypeptide
*PPD*: Protopanaxadiol
*PPT*: Protopanaxatriol
PK: Pharmacokinetics (PK)
QC: Quality control
SC: Schwartz information criterion
UGT: UDP-glucuronosyltransferase
UDPGA: Uridine 5’-diphosphoglucuronosyltransferase
UHPLC-TQD‑MS: Ultra high‑performance liquid chromatography coupled with a triple quadrupole electrospray tandem mass spectrometry
