# Supplementary material for: Multiple circulating alkaloids and saponins from intravenous Kang-Ai injection inhibit human cytochrome P450 and UDP-glucuronosyltransferase isozymes: potential drug–drug interactions
Source: Chin Med. 2020 Jul 6;15:69. doi: 10.1186/s13020-020-00349-3 (PMC7339578; doi:10.1186/s13020-020-00349-3)
Supplement: Supplementary file 1 — Additional file 1: Table S1. Information of unchanged compounds in rats after a 30 min-infusion of Kang-Ai injection (6 mL/kg) by UHPLC/Q-TOF–MS. Table S2. Linear correlation parameters, limits of detection (LODs) and lower limits of quantification (LLOQs) of each circulating compound in rat plasma after a 30 min-infusion of Kang-Ai injection (6 mL/kg). Table S3. Relative standard deviation (RSD) values of intra-day and inter-day precisions, and of sixteen compounds in rat plasma after a 30 min-infusion of Kang-Ai injection (6 mL/kg). Table S4. Matrix effect and extraction recovery of each compound and two internal standard (IS) in rat plasma after a 30 min-infusion of Kang-Ai injection (6 mL/kg). Table S5. Stability results of each compound and internal standard in rat plasma after a 30 min-infusion of Kang-Ai injection (6 mL/kg). Table S6. Linear correlation parameters, LLOQs of twenty analytes in rat urine after a 30 min-infusion of Kang-Ai injection (6 mL/kg). Table S7. Linear correlation parameters, LLOQs of twenty-one analytes in rat bile after a 30 min-infusion of Kang-Ai injection (6 mL/kg). Table S8. The IC50 values of six circulating alkaloids and saponins towards several recombinant CYP and UGT isozymes. Data represent the mean ± standard deviation of triplicate. Fig S1. Effects of different incubation conditions on the metabolism of mephenytoin by human CYP2C19 (mean ± SD, n = 3). (A) Tris buffer concentration; (B) pH values; (C) MgCl2 concentration; (D) NADPH concentration; (E) incubation time; (F) protein concentration. Fig S2. Effects of different incubation conditions on the metabolism of estradiol by UGT1A1. (A) Tris buffer concentration; (B) pH values; (C) MgCl2 concentration; (D) UDPGA concentration; (E) incubation time; (F) protein concentration. Data represent the mean ± standard deviation of triplicate. Fig S3. Chemical structures (A) and content levels (B) of circulating alkaloids, astragalosides and ginsenosides in rats after intravenous Kang [file 13020_2020_349_MOESM1_ESM.docx]

**Additional information**

Multiple circulating alkaloids and saponins from intravenous Kang-Ai injection inhibit human cytochrome P450 and UDP-glucuronosyltransferase isozymes: potential drug-drug interactions

Zifei Qin^1,2,3^, Mengmeng Jia^1,2^, Jing Yang^1,2^*, Han Xing^1,2^, Zhao Yin^1,2^*, Zhihong Yao^3,4^, Xiaojian Zhang^1,2^ and Xinsheng Yao^3,4^

^1^ Department of Pharmacy, the First Affiliated Hospital of Zhengzhou University, Zhengzhou 450052, China;

^2^ Henan Key Laboratory of Precision Clinical Pharmacy, Zhengzhou University, Zhengzhou 450052, China;

^3^ Guangdong Province Key Laboratory of Pharmacodynamic Constituents of TCM and New Drugs Research, Jinan University, Guangzhou 510632, China;

^4^ College of Pharmacy, Jinan University, Guangzhou 510632, China;

*** Corresponding authors**

E-mail: jingyang_0101@163.com (Jing Yang); yinzhao0601@163.com (Zhao Yin);

**Table Lists**

**Table S1.** Information of unchanged compounds in rats after a 30 min-infusion of Kang-Ai injection (6 mL/kg) by UHPLC/Q-TOF-MS.

**Table S2.** Linear correlation parameters, limits of detection (LODs) and lower limits of quantification (LLOQs) of each circulating compound in rat plasma after a 30 min-infusion of Kang-Ai injection (6 mL/kg).

**Table S3.** Relative standard deviation (RSD) values of intra-day and inter-day precisions, and of sixteen compounds in rat plasma after a 30 min-infusion of Kang-Ai injection (6 mL/kg).

**Table S4.** Matrix effect and extraction recovery of each compound and two internal standard (IS) in rat plasma after a 30 min-infusion of Kang-Ai injection (6 mL/kg).

**Table S5.** Stability results of each compound and internal standard in rat plasma after a 30 min-infusion of Kang-Ai injection (6 mL/kg).

**Table S6.** Linear correlation parameters, LLOQs of twenty analytes in rat urine after a 30 min-infusion of Kang-Ai injection (6 mL/kg).

**Table S7.** Linear correlation parameters, LLOQs of twenty-one analytes in rat bile after a 30 min-infusion of Kang-Ai injection (6 mL/kg).

**Table S8.** The IC_50_ values of six circulating alkaloids and saponins towards several recombinant CYP and UGT isozymes. Data represent the mean ± standard deviation of triplicate.

**Figure captions**

**Fig S1. Effects of different incubation conditions on the metabolism of mephenytoin by human CYP2C19 (mean ± SD, n = 3).** (A) Tris buffer concentration; (B) pH values; (C) MgCl_2_ concentration; (D) NADPH concentration; (E) incubation time; (F) protein concentration.

**Fig S2. Effects of different incubation conditions on the metabolism of estradiol by UGT1A1.** (A) Tris buffer concentration; (B) pH values; (C) MgCl_2_ concentration; (D) UDPGA concentration; (E) incubation time; (F) protein concentration. Data represent the mean ± standard deviation of triplicate.

**Fig S3.** Chemical structures (A) and content levels (B) of circulating alkaloids, astragalosides and ginsenosides in rats after intravenous Kang-Ai injection (6 mL/kg). See Supporting Information Table S1 for compounds identification and names.

**Fig S4. Representative MRM chromatograms of sixteen circulating compounds in plasma.** (A) blank plasma, (B) blank plasma spiked with sixteen analytes at LLOQ, (C) rat plasma samples at 0.5 h after an intravenous 30 min-infusion of Kang-Ai injection.

**Fig. S5** **Effects of several circulating compounds towards eight expressed CYP isozymes and three recombinant UGT enzymes.** (A) matrine, (B) ginsenoside Rf, (C) ginsenoside Re, (D) ginsenoside Rd, (E) ginsenoside Rb1; The probe substrates were incubated at 37 ºC in the absence (control, 0 μM) and presence of tested compounds (1, 10, and 100 μM for matrine; 0.1, 1, 10 and 100 μM for astragalosides and ginsenosides). Data represent the mean ± standard deviation of triplicate, (* compared with those of control, * *p* < 0.05).

**Table S1** Information of unchanged compounds in rats after a 30 min-infusion of Kang-Ai injection (6 mL/kg) by UHPLC/Q-TOF-MS

| **ID** | **Compounds** | ***t*_R_**  **(min)** | **Detected**  **ion** | **Error**  **(ppm)** | **Formula** | **Content level**  **(μg/mL)** | **Content level**  **(μM)** | **Sample origin** |
| --- | --- | --- | --- | --- | --- | --- | --- | --- |
| ***Alkaloids*** | | | | | | | | |
| A1 | Matrine | 0.42 | 249.1968 | 0.4 | C_15_H_24_N_2_O | 20.83±9.15 | 84.01±36.89 | plasma/urine/bile |
| A2 | Oxysophocarpine | 0.48 | 263.1761 | 0.4 | C_15_H_22_N_2_O_2_ | 0.65±0.38 | 2.49±1.45 | plasma/urine/bile |
| A3 | Oxymatrine | 0.50 | 265.1922 | 2.3 | C_15_H_24_N_2_O_2_ | 9961.09±524.09 | 37731.41±1985.20 | plasma/urine/bile |
| A4^a^ | 9α-hydroxysophoramine | 1.12 | 261.1607 | 1.5 | C_15_H_20_N_2_O_2_ | 0.10±0.14 | 0.37±0.55 | urine/bile |
| A5^a^ | Baptifoline | 1.64 | 261.1603 | 0 | C_15_H_20_N_2_O_2_ | 1.77±2.01 | 6.82±7.74 | plasma/urine/bile |
| A6^a^ | 9α-hydroxysophocarpine | 0.68 | 263.1763 | 1.1 | C_15_H_22_N_2_O_2_ | 4.49±0.93 | 17.15±3.56 | plasma/urine/bile |
| A7^a^ | Leontalbinine *N*-oxide | 1.36 | 263.1757 | -1.1 | C_15_H_22_N_2_O_2_ | 3.45±1.84 | 13.19±7.04 | plasma/urine/bile |
| A8^a^ | Mamanine | 1.42 | 263.1759 | -0.4 | C_15_H_22_N_2_O_2_ | 0.86±0.68 | 3.26±2.58 | plasma/urine/bile |
| A9^a^ | Sophoranol | 0.32 | 265.1923 | 2.6 | C_15_H_24_N_2_O_2_ | 4.66±0.22 | 17.66±0.84 | plasma/urine/bile |
| ***Astragalosides*** | | | | | | | | |
| B1 | Isoastragaloside IV | 6.52 | 783.4529 | -0.3 | C_41_H_68_O_14_ | 0.69±0.06 | 0.88±0.08 | bile |
| B2 | Astragaloside IV | 7.54 | 783.4529 | -0.3 | C_41_H_68_O_14_ | 71.34±9.17 | 90.99±11.69 | plasma/urine/bile |
| B3 | Astragaloside III | 7.66 | 783.4543 | 1.5 | C_41_H_68_O_14_ | 4.23±0.50 | 5.39±0.64 | plasma/urine/bile |
| B4 | Astragaloside VI | 6.25 | 945.5041 | -1.9 | C_47_H_78_O_19_ | 1.34±0.12 | 1.42±0.12 | urine |
| B5 | Astragaloside V | 7.10 | 945.5051 | -0.8 | C_47_H_78_O_19_ | 2.34±0.13 | 2.47±0.14 | urine |
| B6^a^ | Astragaloside VII | 6.10 | 945.5062 | 0.3 | C_47_H_78_O_19_ | 0.22±0.03 | 0.23±0.03 | urine |
| ***PPT-type ginsenosides*** | | | | | | | | |
| C1 | Ginsenoside Rh1 | 6.87 | 637.4330 | 2.2 | C_36_H_62_O_9_ | 8.58±1.01 | 13.44±1.58 | plasma/urine/bile |
| C2 | Ginsenoside F1 | 7.63 | 637.4307 | -1.4 | C_36_H_62_O_9_ | 0.54±0.20 | 0.85±0.32 | urine/bile |
| C3 | Notoginsenoside R2 | 6.56 | 769.4732 | -0.8 | C_41_H_70_O_13_ | 11.76±0.75 | 15.28±0.98 | plasma/urine/bile |
| C4 | Ginsenoside F3 | 6.89 | 769.4724 | -1.8 | C_41_H_70_O_13_ | 0.33±0.04 | 0.42±0.05 | urine/bile |
| C5 | Ginsenoside Rg2 | 6.86 | 783.4901 | 0.8 | C_42_H_72_O_13_ | 20.70±1.94 | 26.41±2.48 | plasma/urine/bile |
| C6 | Ginsenoside Rg1 | 4.07 | 799.4831 | -1.6 | C_42_H_72_O_14_ | 127.16±8.37 | 158.95±10.47 | plasma/urine/bile |
| C7 | Ginsenoside Rf | 6.35 | 799.4854 | 1.3 | C_42_H_72_O_14_ | 39.57±3.55 | 49.46±4.44 | plasma/urine/bile |
| C8 | Notoginsenoside R1 | 3.25 | 931.5273 | 0.8 | C_47_H_80_O_18_ | 6.75±0.63 | 7.24±0.68 | plasma/urine/bile |
| C9 | Ginsenoside Re | 4.19 | 945.5434 | 1.2 | C_48_H_82_O_18_ | 101.87±5.13 | 107.69±5.42 | plasma/urine/bile |
| C10^a^ | Ginsenoside R1 | 2.86 | 931.5293 | 2.9 | C_47_H_80_O_18_ | 1.13±0.06 | 1.22±0.07 | urine |
| C11^a^ | Ginsenoside Re4 | 3.13 | 931.5276 | 1.1 | C_47_H_80_O_18_ | 1.15±0.08 | 1.23±0.09 | urine |
| C12^a^ | Notoginsenoside R3 | 2.56 | 961.5359 | -1.4 | C_48_H_82_O_19_ | 0.86±0.14 | 0.89±0.14 | urine |
| C13^a^ | 20-Gluco-Ginsenoside Rf | 2.99 | 961.5385 | 1.4 | C_48_H_82_O_19_ | 5.02±0.41 | 5.22±0.43 | urine |
| ***PPD-type ginsenosides*** | | | | | | | | |
| D1 | Ginsenoside F2 | 7.01 | 783.4891 | -0.5 | C_42_H_72_O_13_ | 0.67±0.11 | 0.86±0.14 | bile |
| D2 | Ginsenoside Rd | 8.01 | 945.5428 | 0.5 | C_48_H_82_O_18_ | 7.47±0.84 | 7.89±0.89 | plasma/bile |
| D3 | Ginsenoside Rc | 7.12 | 1077.5857 | 1.1 | C_53_H_90_O_22_ | 10.88±0.69 | 10.09±0.64 | plasma/urine |
| D4 | Ginsenoside Rb2 | 7.42 | 1077.5837 | -0.7 | C_53_H_90_O_22_ | 4.60±0.48 | 4.27±0.45 | Plasma/urine |
| D5 | Ginsenoside Rb1 | 6.88 | 1107.5961 | 0.9 | C_54_H_92_O_23_ | 8.81±0.25 | 7.96±0.23 | plasma/urine |
| ***Other-type ginsenosides*** | | | | | | | | |
| E1 | Ginsenoside Rk3 | 8.60 | 619.4196 | -2.3 | C_36_H_60_O_8_ | 0.97±0.10 | 1.57±0.16 | bile |
| E2 | Ginsenoside Rh4 | 8.66 | 619.4216 | -1.1 | C_36_H_60_O_8_ | 3.19±0.58 | 5.15±0.93 | bile |
| E3 | Ginsenoside Rg6 | 8.51 | 765.4774 | -2 | C_42_H_70_O_12_ | 1.16±0.14 | 1.51±0.18 | bile |
| E4 | Ginsenoside F4 | 8.58 | 765.4794 | 0.7 | C_42_H_70_O_12_ | 1.38±0.22 | 1.80±0.29 | bile |

Note: ^a^ means that these herbal compounds were performed relative quantification.

**Table S2** Linear correlation parameters, limits of detection (LODs) and lower limits of quantification (LLOQs) of each circulating compound in rat plasma after a 30 min-infusion of Kang-Ai injection (6 mL/kg)

| **ID** | **Compound** | **Slope** | **Intercept** | ***r*^2^** | **Linear range (nM)** | **LOD (nM)** | **LLOQ (nM)** |
| --- | --- | --- | --- | --- | --- | --- | --- |
| A1 | Matrine | 0.003962 | 0.000869 | 0.9973 | 10-1000 | 2 | 10 |
|  |  | 0.003927 | 0.080105 | 0.9930 |  |  |  |
|  |  | 0.004319 | 0.081805 | 0.9989 |  |  |  |
| A2 | Oxysophocarpine | 0.008875 | -0.024498 | 0.9964 | 10-1000 | 2 | 10 |
|  |  | 0.008715 | -0.017816 | 0.9967 |  |  |  |
|  |  | 0.008523 | 0.029662 | 0.9970 |  |  |  |
| A3 | Oxymatrine | 0.007640 | 1.540385 | 0.9927 | 40-4000 | 1 | 5 |
|  |  | 0.007707 | 2.002491 | 0.9979 |  |  |  |
|  |  | 0.008253 | 2.361313 | 0.9984 |  |  |  |
| B2 | Astragaloside IV | 0.017732 | -0.098161 | 0.9959 | 40-4000 | 10 | 40 |
|  |  | 0.016803 | 0.075183 | 0.9959 |  |  |  |
|  |  | 0.016632 | 0.144704 | 0.9986 |  |  |  |
| B3 | Astragaloside III | 0.028782 | -0.076207 | 0.9973 | 10-1000 | 2 | 10 |
|  |  | 0.027268 | -0.043682 | 0.9921 |  |  |  |
|  |  | 0.028703 | -0.074203 | 0.9979 |  |  |  |
| C1 | Ginsenoside Rh1 | 0.014249 | 0.053514 | 0.9954 | 10-1000 | 2 | 10 |
|  |  | 0.013718 | -0.030873 | 0.9949 |  |  |  |
|  |  | 0.014471 | -0.083229 | 0.9980 |  |  |  |
| C3 | Notoginsenoside R2 | 0.030279 | 0.024143 | 0.9964 | 10-1000 | 2 | 10 |
|  |  | 0.029031 | 0.010833 | 0.9953 |  |  |  |
|  |  | 0.029423 | -0.091186 | 0.9967 |  |  |  |
| C5 | Ginsenoside Rg2 | 0.027033 | -0.114112 | 0.9959 | 10-1000 | 2 | 10 |
|  |  | 0.025528 | -0.088769 | 0.9963 |  |  |  |
|  |  | 0.026945 | -0.187455 | 0.9992 |  |  |  |
| C6 | Ginsenoside Rg1 | 0.015423 | -0.087963 | 0.9984 | 20-2000 | 5 | 20 |
|  |  | 0.015020 | -0.226994 | 0.9969 |  |  |  |
|  |  | 0.015178 | -0.099755 | 0.9990 |  |  |  |
| C7 | Ginsenoside Rf | 0.012393 | -0.040279 | 0.9989 | 10-1000 | 2 | 10 |
|  |  | 0.010742 | -0.027274 | 0.9900 |  |  |  |
|  |  | 0.011876 | 0.022960 | 0.9991 |  |  |  |
| C8 | Notoginsenoside R1 | 0.015015 | -0.050707 | 0.9921 | 10-1000 | 2 | 10 |
|  |  | 0.014038 | -0.026616 | 0.9914 |  |  |  |
|  |  | 0.014855 | -0.087305 | 0.9990 |  |  |  |
| C9 | Ginsenoside Re | 0.020928 | -0.066897 | 0.9930 | 20-2000 | 5 | 20 |
|  |  | 0.019858 | -0.013526 | 0.9952 |  |  |  |
|  |  | 0.020556 | -0.003369 | 0.9986 |  |  |  |
| D2 | Ginsenoside Rd | 0.022600 | 0.054201 | 0.9994 | 10-1000 | 2 | 10 |
|  |  | 0.022299 | -0.055895 | 0.9990 |  |  |  |
|  |  | 0.022243 | -0.090317 | 0.9988 |  |  |  |
| D3 | Ginsenoside Rc | 0.012375 | -0.041648 | 0.9959 | 10-1000 | 2 | 10 |
|  |  | 0.012554 | -0.048137 | 0.9973 |  |  |  |
|  |  | 0.012284 | 0.048605 | 0.9960 |  |  |  |
| D4 | Ginsenoside Rb2 | 0.015032 | -0.004867 | 0.9978 | 10-1000 | 2 | 10 |
|  |  | 0.016223 | -0.044610 | 0.9946 |  |  |  |
|  |  | 0.015793 | -0.012628 | 0.9960 |  |  |  |
| D5 | Ginsenoside Rb1 | 0.010137 | 0.005106 | 0.9926 | 10-1000 | 2 | 10 |
|  |  | 0.009313 | 0.005881 | 0.9920 |  |  |  |
|  |  | 0.010005 | 0.059154 | 0.9964 |  |  |  |

**Table S3** Relative standard deviation (RSD) values of intra-day and inter-day precisions, and of sixteen compounds in rat plasma after a 30 min-infusion of Kang-Ai injection (6 mL/kg)

| **ID** | **Compound** | **Concentration**  **(nM)** | **Intra-day (n = 6)** | | | **Inter-day (n = 18)** | | |
| --- | --- | --- | --- | --- | --- | --- | --- | --- |
|  |  |  | **Mean ± SD (nM)** | **RE (%)** | **RSD (%)** | **Mean ± SD (nM)** | **RE (%)** | **RSD (%)** |
| A1 | Matrine | 20 | 21.24±0.70 | 10.02 | 3.31 | 20.04±1.65 | -11.91 | 8.24 |
|  |  | 100 | 99.90±8.33 | -9.66 | 8.34 | 99.55±7.95 | -12.66 | 7.98 |
|  |  | 400 | 376.89±20.37 | -10.64 | 5.41 | 403.40±26.37 | 9.30 | 6.54 |
| A2 | Oxysophocarpine | 20 | 20.42±1.97 | 10.14 | 9.65 | 21.12±1.36 | 11.24 | 6.44 |
|  |  | 100 | 106.35±5.97 | 12.90 | 5.62 | 102.41±6.44 | 8.82 | 6.29 |
|  |  | 400 | 418.32±37.52 | 11.61 | 8.97 | 412.11±29.36 | 10.58 | 7.13 |
| A3 | Oxymatrine | 80 | 80.97±2.60 | 3.70 | 3.21 | 81.67±1.94 | 4.52 | 2.38 |
|  |  | 400 | 378.61±37.48 | -11.54 | 9.90 | 401.31±30.52 | 10.93 | 7.61 |
|  |  | 1600 | 1542.37±117.35 | -8.77 | 7.61 | 1632.84±118.74 | 11.13 | 7.27 |
| B2 | Astragaloside IV | 80 | 78.56±5.91 | -9.09 | 7.52 | 87.72±4.79 | 10.68 | 5.79 |
|  |  | 400 | 399.91±39.41 | 10.13 | 9.85 | 400.11±24.83 | 6.21 | 9.55 |
|  |  | 1600 | 1590.81±108.69 | 7.25 | 6.83 | 1583.58±84.92 | -9.85 | 5.36 |
| B3 | Astragaloside III | 20 | 20.19±1.88 | -9.43 | 9.31 | 20.70±1.52 | 12.51 | 7.34 |
|  |  | 100 | 104.01±10.15 | 10.83 | 9.76 | 102.10±8.64 | 8.85 | 8.46 |
|  |  | 400 | 393.50±48.50 | 12.37 | 12.33 | 402.15±30.93 | -8.49 | 7.69 |
| C1 | Ginsenoside Rh1 | 20 | 19.27±1.02 | -8.06 | 5.30 | 19.93±1.70 | -12.36 | 8.51 |
|  |  | 100 | 97.39±9.26 | -9.56 | 9.51 | 103.36±8.63 | 12.51 | 8.35 |
|  |  | 400 | 397.65±27.96 | 7.48 | 7.03 | 412.11±26.68 | 13.29 | 6.47 |
| C3 | Notoginsenoside R2 | 20 | 19.84±1.41 | -8.76 | 7.13 | 19.44±1.11 | -9.63 | 5.71 |
|  |  | 100 | 102.18±12.54 | 12.01 | 12.27 | 101.31±7.55 | -11.94 | 7.45 |
|  |  | 400 | 397.62±31.01 | 8.11 | 7.80 | 411.91±30.98 | 12.30 | 7.52 |
| C5 | Ginsenoside Rg2 | 20 | 19.89±1.62 | -9.02 | 8.14 | 21.00±1.49 | 13.59 | 7.09 |
|  |  | 100 | 97.40±7.43 | -8.63 | 7.63 | 102.22±7.31 | 9.80 | 7.15 |
|  |  | 400 | 386.01±36.48 | -12.84 | 9.45 | 406.26±33.06 | 13.19 | 8.14 |
| C6 | Ginsenoside Rg1 | 40 | 38.68±2.17 | -6.49 | 5.62 | 39.53±2.30 | -8.20 | 5.81 |
|  |  | 200 | 197.91±18.15 | 9.42 | 9.17 | 191.65±10.60 | -8.79 | 5.53 |
|  |  | 800 | 800.76±47.28 | 6.92 | 5.90 | 797.12±41.36 | 8.37 | 5.19 |
| C7 | Ginsenoside Rf | 20 | 18.94±1.90 | -14.30 | 10.02 | 20.28±1.54 | 10.69 | 7.58 |
|  |  | 100 | 93.88±11.46 | -12.86 | 12.20 | 98.25±8.94 | -12.61 | 9.10 |
|  |  | 400 | 396.08±9.29 | -2.59 | 2.35 | 414.88±16.49 | 9.48 | 3.98 |
| C8 | Notoginsenoside R1 | 20 | 20.43±2.58 | 12.94 | 12.64 | 20.02±1.73 | -12.15 | 8.63 |
|  |  | 100 | 97.59±9.61 | -10.47 | 9.84 | 97.57±6.13 | 8.22 | 6.28 |
|  |  | 400 | 386.42±35.58 | -11.47 | 9.21 | 403.65±32.53 | 10.28 | 8.06 |
| C9 | Ginsenoside Re | 40 | 39.72±2.58 | 6.57 | 6.51 | 40.65±2.97 | 13.66 | 7.24 |
|  |  | 200 | 191.74±22.06 | -11.75 | 11.51 | 193.86±15.81 | -10.90 | 8.16 |
|  |  | 800 | 794.63±63.20 | 8.08 | 7.95 | 810.97±49.77 | 11.21 | 6.14 |
| D2 | Ginsenoside Rd | 20 | 20.28±1.51 | 8.11 | 7.47 | 21.02±1.05 | 9.64 | 5.02 |
|  |  | 100 | 105.71±6.23 | 12.82 | 5.89 | 105.05±4.79 | 8.40 | 4.56 |
|  |  | 400 | 385.33±21.61 | -9.83 | 5.61 | 403.91±24.74 | 11.29 | 6.13 |
| D3 | Ginsenoside Rc | 20 | 20.48±2.02 | 10.01 | 9.86 | 20.01±1.49 | -10.06 | 7.43 |
|  |  | 100 | 98.25±8.32 | -11.34 | 8.46 | 100.10±5.18 | 4.91 | 5.17 |
|  |  | 400 | 392.57±32.04 | 7.38 | 8.16 | 402.41±21.80 | -6.80 | 5.42 |
| D4 | Ginsenoside Rb2 | 20 | 20.46±1.99 | 11.08 | 9.73 | 20.21±1.44 | -9.16 | 7.10 |
|  |  | 100 | 96.60±4.40 | -5.90 | 4.56 | 103.49±6.58 | 12.30 | 6.36 |
|  |  | 400 | 395.08±36.11 | 9.19 | 9.14 | 403.95±28.23 | 10.69 | 6.99 |
| D5 | Ginsenoside Rb1 | 20 | 20.41±1056 | 8.19 | 7.65 | 20.17±1.16 | -8.57 | 5.76 |
|  |  | 100 | 103.96±8.92 | 11.38 | 8.58 | 104.04±7.28 | 12.80 | 7.00 |
|  |  | 400 | 418.71±31.55 | 9.81 | 7.54 | 411.59±25.33 | 8.99 | 6.15 |

**Table S4** Matrix effect and extraction recovery of each compound and two internal standard (IS) in rat plasma after a 30 min-infusion of Kang-Ai injection (6 mL/kg)

| **ID** | **Compound** | **Concentration (nM)** | **Matrix effect (%)** | **RSD (%)** | **Extraction recovery (%)** | **RSD (%)** |
| --- | --- | --- | --- | --- | --- | --- |
| A1 | Matrine | 20 | 97.15±8.32 | 8.56 | 99.96±8.70 | 8.70 |
|  |  | 100 | 100.69±10.94 | 10.86 | 102.28±11.06 | 10.82 |
|  |  | 400 | 104.25±6.38 | 6.12 | 86.91±1.81 | 2.09 |
| A2 | Oxysophocarpine | 20 | 103.61±8.62 | 8.32 | 100.26±10.57 | 10.54 |
|  |  | 100 | 100.35±10.48 | 10.44 | 108.98±6.07 | 5.57 |
|  |  | 400 | 105.13±4.79 | 4.56 | 88.46±2.91 | 3.29 |
| A3 | Oxymatrine | 80 | 100.75±5.86 | 5.82 | 105.17±8.62 | 8.19 |
|  |  | 400 | 100.93±1.97 | 1.95 | 105.83±6.93 | 6.55 |
|  |  | 1600 | 108.18±3.90 | 3.61 | 89.64±3.30 | 3.68 |
| B2 | Astragaloside IV | 80 | 101.61±13.15 | 12.94 | 91.43±5.07 | 5.54 |
|  |  | 400 | 108.39±3.55 | 3.28 | 84.60±3.05 | 3.60 |
|  |  | 1600 | 93.43±4.16 | 4.45 | 93.34±0.98 | 1.05 |
| B3 | Astragaloside III | 20 | 105.55±9.80 | 9.29 | 102.92±3.04 | 2.95 |
|  |  | 100 | 99.87±10.68 | 10.69 | 83.63±1.03 | 1.23 |
|  |  | 400 | 98.52±4.98 | 5.06 | 92.25±3.62 | 3.93 |
| C1 | Ginsenoside Rh1 | 20 | 100.13±5.48 | 5.47 | 98.82±10.66 | 11.25 |
|  |  | 100 | 108.26±3.21 | 2.96 | 87.97±1.31 | 1.49 |
|  |  | 400 | 104.51±2.44 | 2.34 | 90.40±3.23 | 3.57 |
| C3 | Notoginsenoside R2 | 20 | 99.09±6.68 | 6.74 | 92.77±9.36 | 10.09 |
|  |  | 100 | 103.64±3.56 | 3.44 | 89.16±1.19 | 1.33 |
|  |  | 400 | 96.08±4.21 | 4.38 | 98.81±6.38 | 6.46 |
| C5 | Ginsenoside Rg2 | 20 | 104.39±10.38 | 9.94 | 99.00±5.21 | 5.26 |
|  |  | 100 | 106.72±9.00 | 8.43 | 94.95±13.51 | 14.23 |
|  |  | 400 | 103.05±9.39 | 9.11 | 94.99±1.78 | 1.87 |
| C6 | Ginsenoside Rg1 | 40 | 92.06±6.88 | 7.48 | 98.42±7.75 | 7.88 |
|  |  | 200 | 94.22±8.55 | 9.08 | 86.28±6.89 | 7.98 |
|  |  | 800 | 98.43±9.35 | 9.50 | 92.14±11.01 | 11.95 |
| C7 | Ginsenoside Rf | 20 | 101.24±7.82 | 7.72 | 84.79±3.31 | 3.90 |
|  |  | 100 | 105.85±6.31 | 5.96 | 93.93±4.56 | 4.85 |
|  |  | 400 | 108.29±7.45 | 6.88 | 88.02±5.68 | 6.46 |
| C8 | Notoginsenoside R1 | 20 | 104.58±14.79 | 14.14 | 99.68±10.51 | 10.54 |
|  |  | 100 | 102.67±1.31 | 1.28 | 84.71±2.17 | 2.57 |
|  |  | 400 | 102.71±8.77 | 8.54 | 87.44±3.89 | 4.45 |
| C9 | Ginsenoside Re | 40 | 97.71±3.91 | 4.00 | 84.68±2.98 | 3.52 |
|  |  | 200 | 110.45±2.83 | 2.56 | 82.85±2.10 | 2.54 |
|  |  | 800 | 103.82±5.13 | 4.94 | 90.05±3.61 | 4.00 |
| D2 | Ginsenoside Rd | 20 | 105.06±8.87 | 8.44 | 99.33±9.36 | 9.42 |
|  |  | 100 | 93.90±3.23 | 3.44 | 102.49±4.04 | 3.94 |
|  |  | 400 | 96.14±5.83 | 6.06 | 95.48±3.58 | 3.75 |
| D3 | Ginsenoside Rc | 20 | 103.80±13.18 | 12.70 | 88.57±7.91 | 8.94 |
|  |  | 100 | 98.95±12.51 | 12.64 | 102.68±2.46 | 2.40 |
|  |  | 400 | 100.09±4.57 | 4.57 | 87.64±6.22 | 7.09 |
| D4 | Ginsenoside Rb2 | 20 | 109.13±3.59 | 3.29 | 89.01±7.64 | 8.58 |
|  |  | 100 | 105.95±4.49 | 4.24 | 80.84±2.14 | 2.65 |
|  |  | 400 | 100.09±1.91 | 1.91 | 90.94±4.55 | 5.01 |
| D5 | Ginsenoside Rb1 | 20 | 97.22±8.04 | 8.27 | 96.18±14.17 | 14.73 |
|  |  | 100 | 99.16±11.65 | 11.75 | 89.29±3.21 | 3.59 |
|  |  | 400 | 105.21±11.86 | 11.27 | 90.85±7.16 | 7.88 |
| IS-1 | Olanzapine-d3 | 20 | 100.29±11.45 | 11.42 | 80.54±1.70 | 2.11 |
| IS-2 | Mycophenolic acid-d3 | 1000 | 109.12±3.42 | 3.13 | 83.60±4.34 | 5.19 |

**Table S5** Stability results of each compound and internal standard in rat plasma after a 30 min-infusion of Kang-Ai injection (6 mL/kg)

| **ID** | **Conc.**  **(nM)** | **Short-term stability at room temperature for 6 h** | | | **Extracted samples at 8 °C for 18 h** | | | **Three freeze-thaw cycles (-80 °C ↔ 23 °C)** | | | **At -80 °C for 48 h** | | |
| --- | --- | --- | --- | --- | --- | --- | --- | --- | --- | --- | --- | --- | --- |
|  |  | **Mean±SD**  (nM) | **RE**  **(%)** | **RSD**  **(%)** | **Mean±SD**  (nM) | **RE**  **(%)** | **RSD**  **(%)** | **Mean±SD**  (nM) | **RE**  **(%)** | **RSD**  **(%)** | **Mean±SD**  (nM) | **RE**  **(%)** | **RSD**  **(%)** |
| A1 | 20 | 20.84±0.75 | 8.05 | 3.60 | 21.75±0.99 | 13.23 | 4.57 | 20.11±1.97 | -9.75 | 9.82 | 21.33±1.64 | 10.69 | 7.67 |
|  | 100 | 100.30±2.84 | 3.05 | 2.83 | 95.18±2.64 | -7.17 | 2.78 | 104.44±12.01 | 14.16 | 11.50 | 106.76±6.22 | 13.15 | 5.83 |
|  | 400 | 420.24±21.99 | 11.33 | 5.23 | 402.13±26.43 | 7.87 | 6.57 | 415.19±9.89 | 5.94 | 2.38 | 394.46±17.47 | -6.06 | 4.43 |
| A2 | 20 | 20.94±1.33 | 12.22 | 6.34 | 22.58±0.11 | 13.22 | 0.49 | 22.63±0.15 | 13.81 | 0.68 | 21.90±0.32 | 10.73 | 1.47 |
|  | 100 | 103.12±8.16 | 12.17 | 7.96 | 108.59±6.59 | 14.25 | 6.07 | 103.40±9.41 | 12.06 | 9.10 | 112.64±0.85 | 13.54 | 0.75 |
|  | 400 | 439.31±14.14 | 12.94 | 3.22 | 432.22±7.29 | 9.98 | 1.69 | 418.53±37.48 | 14.91 | 8.96 | 437.15±14.08 | 11.61 | 3.22 |
| A3 | 80 | 81.69±3.23 | 6.63 | 3.96 | 76.52±2.34 | -6.93 | 3.05 | 82.92±3.13 | 8.11 | 3.78 | 83.89±6.74 | 13.90 | 8.03 |
|  | 400 | 391.75±44.05 | 10.53 | 11.24 | 410.83±21.99 | 7.99 | 5.35 | 406.54±20.44 | 7.52 | 5.03 | 422.79±10.74 | 8.62 | 2.54 |
|  | 1600 | 1629.42±112.91 | 8.70 | 6.93 | 1553.29±127.31 | -9.16 | 8.20 | 1588.35±117.83 | 7.77 | 7.42 | 1578.58±168.35 | 10.74 | 10.66 |
| B2 | 80 | 82.35±9.84 | -11.26 | 11.95 | 80.76±10.01 | -13.07 | 12.39 | 88.56±1.31 | 12.35 | 1.48 | 87.06±0.84 | 9.91 | 0.96 |
|  | 400 | 361.42±15.40 | -13.36 | 4.26 | 376.72±29.81 | -10.44 | 7.91 | 385.93±32.19 | -12.56 | 8.34 | 378.90±6.78 | -7.16 | 1.79 |
|  | 1600 | 1463.38±60.14 | -11.64 | 4.11 | 1574.79±151.38 | -11.93 | 9.61 | 1434.32±47.18 | -13.10 | 3.29 | 1507.16±152.79 | -12.08 | 10.14 |
| B3 | 20 | 19.47±1.70 | -12.31 | 8.72 | 17.98±1.29 | -14.29 | 7.18 | 19.24±0.59 | -6.46 | 3.04 | 19.85±0.45 | -3.31 | 2.28 |
|  | 100 | 91.15±3.72 | -13.15 | 4.08 | 99.17±12.67 | 13.54 | 12.78 | 103.49±8.05 | 9.29 | 7.77 | 89.68±4.10 | -13.24 | 4.57 |
|  | 400 | 348.69±3.47 | -13.71 | 1.00 | 400.78±32.83 | -8.93 | 8.19 | 387.44±17.00 | -6.84 | 4.39 | 431.79±15.31 | 12.29 | 3.55 |
| C1 | 20 | 20.81±2.57 | 13.31 | 12.37 | 20.79±2.88 | -12.72 | 13.87 | 20.14±2.50 | -13.10 | 12.40 | 22.38±0.33 | 13.76 | 1.46 |
|  | 100 | 92.25±8.56 | -13.70 | 9.28 | 100.20±11.08 | 12.81 | 11.06 | 99.91±9.81 | -10.86 | 9.82 | 88.26±1.17 | -13.03 | 1.32 |
|  | 400 | 376.44±26.61 | -12.62 | 7.07 | 424.42±36.39 | 12.32 | 8.57 | 370.01±38.18 | -13.77 | 10.32 | 379.88±25.01 | -9.26 | 6.58 |
| C3 | 20 | 20.21±2.65 | -13.56 | 13.12 | 18.64±1.82 | -13.82 | 9.75 | 18.73±0.89 | -9.00 | 4.77 | 20.45±1.00 | 7.72 | 4.89 |
|  | 100 | 89.70±2.05 | -11.58 | 2.28 | 97.64±10.65 | -10.93 | 10.91 | 98.27±11.41 | 10.38 | 11.61 | 94.78±7.04 | -10.60 | 7.42 |
|  | 400 | 361.17±15.30 | -12.36 | 4.23 | 407.84±38.17 | -9.03 | 9.36 | 391.16±22.13 | -5.92 | 5.66 | 405.34±28.21 | 9.16 | 6.96 |
| C5 | 20 | 19.68±2.51 | 12.85 | 12.74 | 21.07±0.87 | 8.32 | 4.11 | 21.26±1.22 | 11.13 | 5.72 | 19.84±1.04 | -6.64 | 5.23 |
|  | 100 | 87.52±0.79 | -12.97 | 0.91 | 98.05±6.29 | -8.46 | 6.42 | 104.41±11.32 | 12.73 | 10.84 | 96.95±8.62 | -8.82 | 8.89 |
|  | 400 | 361.60±11.46 | -11.77 | 3.17 | 404.47±42.95 | -10.83 | 10.62 | 374.05±38.03 | -12.71 | 10.17 | 410.70±32.70 | 8.22 | 7.96 |
| C6 | 40 | 38.60±1.14 | -6.74 | 2.95 | 35.69±0.56 | -12.18 | 1.57 | 39.64±2.41 | -7.72 | 6.08 | 37.29±1.37 | -10.20 | 3.66 |
|  | 200 | 204.22±5.64 | 4.83 | 2.76 | 197.72±17.37 | 8.81 | 8.79 | 189.03±9.20 | -10.01 | 4.87 | 209.85±12.74 | 10.34 | 6.07 |
|  | 800 | 737.30±34.89 | -11.11 | 4.73 | 826.40±51.24 | 10.42 | 6.20 | 739.78±39.17 | -11.08 | 5.29 | 800.01±77.30 | 11.14 | 9.66 |
| C7 | 20 | 20.44±2.11 | 11.15 | 10.31 | 19.73±2.64 | 13.56 | 13.37 | 22.07±0.56 | 13.46 | 2.52 | 21.60±0.37 | 9.41 | 1.72 |
|  | 100 | 96.37±9.64 | -10.30 | 10.0 | 93.83±2.15 | -8.56 | 2.29 | 101.34±13.26 | -13.01 | 13.08 | 100.58±11.11 | 13.37 | 11.05 |
|  | 400 | 357.16±4.75 | -11.69 | 1.33 | 392.21±39.26 | -12.43 | 10.01 | 398.07±39.06 | -10.52 | 9.81 | 428.42±28.06 | 14.40 | 6.55 |
| C8 | 20 | 17.74±0.16 | -12.08 | 0.89 | 19.67±2.39 | -11.85 | 12.13 | 21.51±1.68 | 12.79 | 7.81 | 20.74±0.96 | 8.27 | 4.64 |
|  | 100 | 92.24±4.21 | -10.97 | 4.57 | 97.66±12.82 | 12.46 | 13.13 | 97.29±6.47 | -10.11 | 6.65 | 98.21±9.98 | 9.58 | 10.17 |
|  | 400 | 357.29±9.94 | -13.09 | 2.78 | 402.54±33.33 | 9.82 | 8.28 | 379.04±38.85 | -14.53 | 10.25 | 413.01±31.69 | 11.76 | 7.67 |
| C9 | 40 | 42.26±4.77 | 12.91 | 11.28 | 39.68±3.10 | -8.46 | 7.82 | 39.18±1.53 | -5.32 | 3.90 | 37.07±2.41 | -12.37 | 6.50 |
|  | 200 | 175.60±2.61 | -13.13 | 1.49 | 193.91±26.18 | -12.50 | 13.50 | 205.17±19.51 | 11.83 | 9.51 | 181.77±7.39 | -12.74 | 4.07 |
|  | 800 | 724.25±14.79 | -11.32 | 2.04 | 827.44±80.86 | 12.75 | 9.77 | 818.48±84.93 | 12.80 | 10.38 | 836.79±51.65 | 10.56 | 6.17 |
| D2 | 20 | 20.41±1.88 | -8.78 | 9.22 | 21.52±1.04 | 11.29 | 4.82 | 21.38±0.85 | 11.74 | 3.96 | 20.86±2.70 | 13.40 | 12.93 |
|  | 100 | 96.61±5.06 | -8.93 | 5.24 | 93.92±4.07 | -9.39 | 4.34 | 101.66±11.99 | 13.24 | 11.79 | 94.76±3.45 | -9.18 | 3.64 |
|  | 400 | 362.78±5.91 | -10.80 | 1.63 | 411.97±35.80 | 9.32 | 8.69 | 397.28±35.89 | 9.66 | 9.03 | 419.98±19.52 | 10.54 | 4.65 |
| D3 | 20 | 21.00±1.54 | 10.56 | 7.34 | 21.26±0.64 | 9.82 | 3.00 | 22.31±0.31 | 12.70 | 1.38 | 21.79±0.54 | 11.90 | 2.47 |
|  | 100 | 94.62±9.91 | -13.45 | 10.48 | 91.90±3.48 | -12.09 | 3.78 | 107.11±6.52 | 11.88 | 6.08 | 92.14±3.15 | -10.73 | 3.42 |
|  | 400 | 366.80±3.85 | -9.03 | 1.05 | 432.26±28.61 | 12.97 | 6.62 | 413.15±9.39 | 5.12 | 2.27 | 421.69±7.21 | 7.06 | 1.71 |
| D4 | 20 | 20.28±1.99 | 10.37 | 9.82 | 19.58±2.21 | -12.48 | 11.30 | 20.89±1.50 | 11.32 | 7.17 | 20.39±1.54 | -6.96 | 7.56 |
|  | 100 | 88.84±2.61 | -13.48 | 2.94 | 96.04±12.11 | -10.99 | 12.61 | 104.61±9.20 | 10.55 | 8.80 | 95.34±8.02 | -12.47 | 8.41 |
|  | 400 | 352.24±2.93 | -12.50 | 0.83 | 434.41±11.65 | 11.01 | 2.68 | 440.44±2.62 | 13.72 | 2.87 | 423.10±46.59 | 12.64 | 11.01 |
| D5 | 20 | 20.30±1.81 | 9.00 | 8.92 | 17.96±0.32 | -11.74 | 1.77 | 19.99±1.88 | -10.85 | 9.39 | 20.64±1.63 | 8.70 | 7.91 |
|  | 100 | 91.65±3.42 | -12.30 | 3.73 | 101.02±12.87 | -13.83 | 12.74 | 108.29±6.26 | 12.24 | 5.78 | 94.32±8.04 | -13.46 | 8.52 |
|  | 400 | 388.96±18.81 | -6.00 | 4.84 | 409.55±29.87 | 9.12 | 7.29 | 441.95±3.33 | 11.44 | 0.75 | 428.49±29.97 | 12.25 | 7.00 |

Note. See Table S1 for the compounds’ ID and names.

**Table S6** Linear correlation parameters, LLOQs of twenty analytes in rat urine after a 30 min-infusion of Kang-Ai injection (6 mL/kg)

| **ID** | **Compound** | **Slope** | **Intercept** | ***r*^2^** | **Linear range (nM)** | **LLOQ (nM)** |
| --- | --- | --- | --- | --- | --- | --- |
| A1 | Matrine | 0.003932 | 0.061829 | 0.9920 | 20-2000 | 10 |
| A2 | Oxysophocarpine | 0.004823 | 0.440337 | 0.9944 | 50-5000 | 10 |
| A3 | Oxymatrine | 0.005742 | 2.800294 | 0.9984 | 80-8000 | 5 |
| B2 | Astragaloside IV | 0.009049 | -0.451304 | 0.9957 | 200-20000 | 40 |
| B3 | Astragaloside III | 0.010215 | -0.053452 | 0.9976 | 10-1000 | 10 |
| B4 | Astragaloside VI | 0.010611 | 0.022832 | 0.9975 | 10-1000 | 10 |
| B5 | Astragaloside V | 0.009275 | -0.011719 | 0.9948 | 10-1000 | 10 |
| C1 | Ginsenoside Rh1 | 0.006969 | 0.018261 | 0.9928 | 10-1000 | 10 |
| C2 | Ginsenoside F1 | 0.005376 | 0.003825 | 0.9978 | 10-1000 | 10 |
| C3 | Notoginsenoside R2 | 0.008949 | 0.002436 | 0.9962 | 10-1000 | 10 |
| C4 | Ginsenoside F3 | 0.005713 | 0.008059 | 0.9906 | 10-1000 | 10 |
| C5 | Ginsenoside Rg2 | 0.010635 | -0.012429 | 0.9973 | 10-1000 | 10 |
| C6 | Ginsenoside Rg1 | 0.005081 | -0.580012 | 0.9932 | 400-40000 | 20 |
| C7 | Ginsenoside Rf | 0.007660 | -0.125340 | 0.9969 | 40-4000 | 10 |
| C8 | Notoginsenoside R1 | 0.007740 | -0.152998 | 0.9982 | 40-4000 | 10 |
| C9 | Ginsenoside Re | 0.008792 | -0.410486 | 0.9939 | 200-20000 | 20 |
| D2 | Ginsenoside Rd | 0.011737 | 0.001810 | 0.9953 | 10-1000 | 10 |
| D3 | Ginsenoside Rc | 0.007948 | -0.001516 | 0.9991 | 10-1000 | 10 |
| D4 | Ginsenoside Rb2 | 0.005475 | 0.018608 | 0.9939 | 10-1000 | 10 |
| D5 | Ginsenoside Rb1 | 0.005333 | 0.099203 | 0.9988 | 10-1000 | 10 |

**Table S7** Linear correlation parameters, LLOQs of twenty-one analytes in rat bile after a 30 min-infusion of Kang-Ai injection (6 mL/kg)

| **ID** | **Compound** | **Slope** | **Intercept** | ***r*^2^** | **Linear range (nM)** | **LLOQ (nM)** |
| --- | --- | --- | --- | --- | --- | --- |
| A1 | Matrine | 0.003844 | 0.441549 | 0.9986 | 40-4000 | 10 |
| A2 | Oxysophocarpine | 0.004836 | 0.157156 | 0.9958 | 20-2000 | 10 |
| A3 | Oxymatrine | 0.004311 | 3.568092 | 0.9947 | 200-20000 | 5 |
| B1 | Isoastragaloside IV | 0.011135 | -0.076077 | 0.9991 | 10-1000 | 10 |
| B2 | Astragaloside IV | 0.010981 | -0.456010 | 0.9968 | 200-20000 | 40 |
| B3 | Astragaloside III | 0.012957 | -0.244007 | 0.9901 | 40-4000 | 10 |
| C1 | Ginsenoside Rh1 | 0.007545 | 0.251923 | 0.9941 | 40-4000 | 10 |
| C2 | Ginsenoside F1 | 0.009507 | 0.257863 | 0.9985 | 10-1000 | 10 |
| C3 | Notoginsenoside R2 | 0.009662 | -0.133363 | 0.9972 | 100-10000 | 10 |
| C4 | Ginsenoside F3 | 0.007304 | 0.059130 | 0.9928 | 10-1000 | 10 |
| C5 | Ginsenoside Rg2 | 0.010221 | -2.396374 | 0.9991 | 200-20000 | 10 |
| C6 | Ginsenoside Rg1 | 0.006976 | 0.713827 | 0.9983 | 400-40000 | 20 |
| C7 | Ginsenoside Rf | 0.008270 | -0.239619 | 0.9967 | 200-20000 | 10 |
| C8 | Notoginsenoside R1 | 0.008317 | 0.108672 | 0.9967 | 20-2000 | 10 |
| C9 | Ginsenoside Re | 0.009921 | 0.285275 | 0.9976 | 400-40000 | 20 |
| D1 | Ginsenoside F2 | 0.008013 | 0.094963 | 0.9988 | 10-1000 | 10 |
| D2 | Ginsenoside Rd | 0.014398 | -0.065688 | 0.9977 | 10-1000 | 10 |
| E1 | Ginsenoside Rk3 | 0.009203 | 0.191908 | 0.9955 | 10-1000 | 10 |
| E2 | Ginsenoside Rh4 | 0.001252 | 0.109971 | 0.9952 | 40-4000 | 20 |
| E3 | Ginsenoside Rg6 | 0.008053 | 0.056766 | 0.9985 | 10-1000 | 10 |
| E4 | Ginsenoside F4 | 0.006240 | 0.425566 | 0.9922 | 10-1000 | 10 |

**Table S8** The IC_50_ values of six circulating alkaloids and saponins towards several recombinant CYP and UGT isozymes. Data represent the mean ± standard deviation of triplicate.

| **Compounds** | **CYP1A2** | **CYP2A6** | **CYP2B6** | **CYP2C8** | **CYP2C9** | **CYP2C19** | **CYP2E1** | **CYP3A4** | **UGT1A1** | **UGT1A9** | **UGT2B7** |
| --- | --- | --- | --- | --- | --- | --- | --- | --- | --- | --- | --- |
| Matrine (A1) | NI | NI | NI | NI | NI | NI | NI | NI | NI | NI | NI |
| Oxymatrine (A3) | NI | NI | NI | NI | NI | NI | NI | NI | NI | NI | NI |
| Astragaloside IV (B2) | NI | NI | NI | NI | 65.0±38.7 | NI | NI | NI | NI | NI | NI |
| Ginsenoside Rg1 (C6) | NI | NI | NI | NI | NI | NI | NI | NI | 92.2±40.0 | NI | NI |
| Ginsenoside Rf (C7) | NI | NI | NI | NI | NI | NI | NI | NI | >100 | NI | >100 |
| Ginsenoside Re (C9) | NI | NI | NI | 24.0±10.7 | NI | NI | NI | NI | >100 | NI | >100 |
| Ginsenoside Rd (D2) | >100 | NI | 20.2±12.4 | NI | >100 | >100 | NI | 41.0±25.8 | NI | NI | >100 |
| Ginsenoside Rc (D3) | NI | NI | NI | NI | Activation | NI | NI | NI | >100 | 29.0±12.7 | NI |
| Ginsenoside Rb1 (D5) | NI | NI | NI | NI | 8.8±3.2 | >100 | NI | >100 | NI | >100 | >100 |

Note: NI mean the herbal compounds have no inhibitory effects towards the tested CYP and UGT isozymes *in vitro*.





**Figure S1**





**Figure S2**


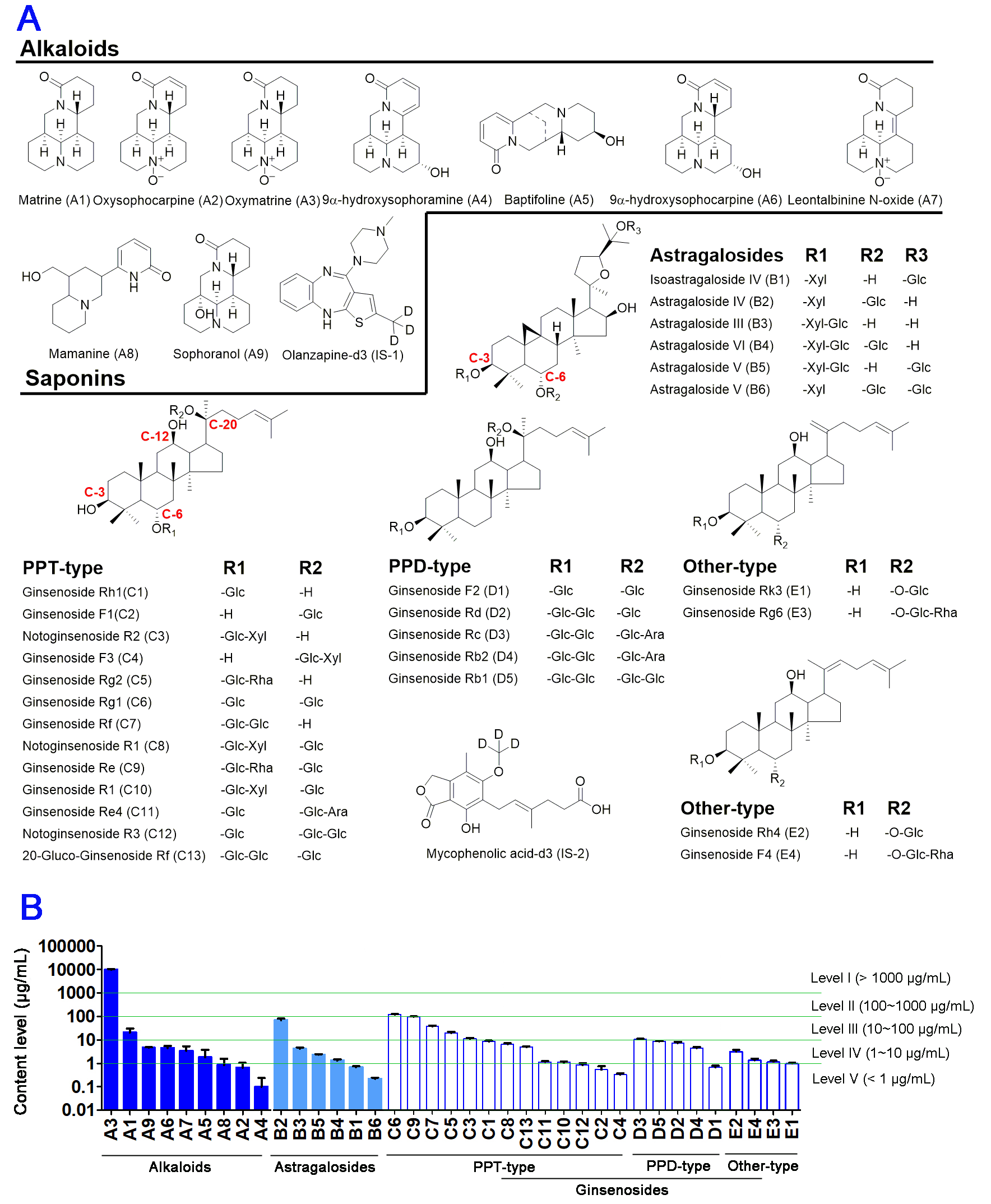


**Figure S3**


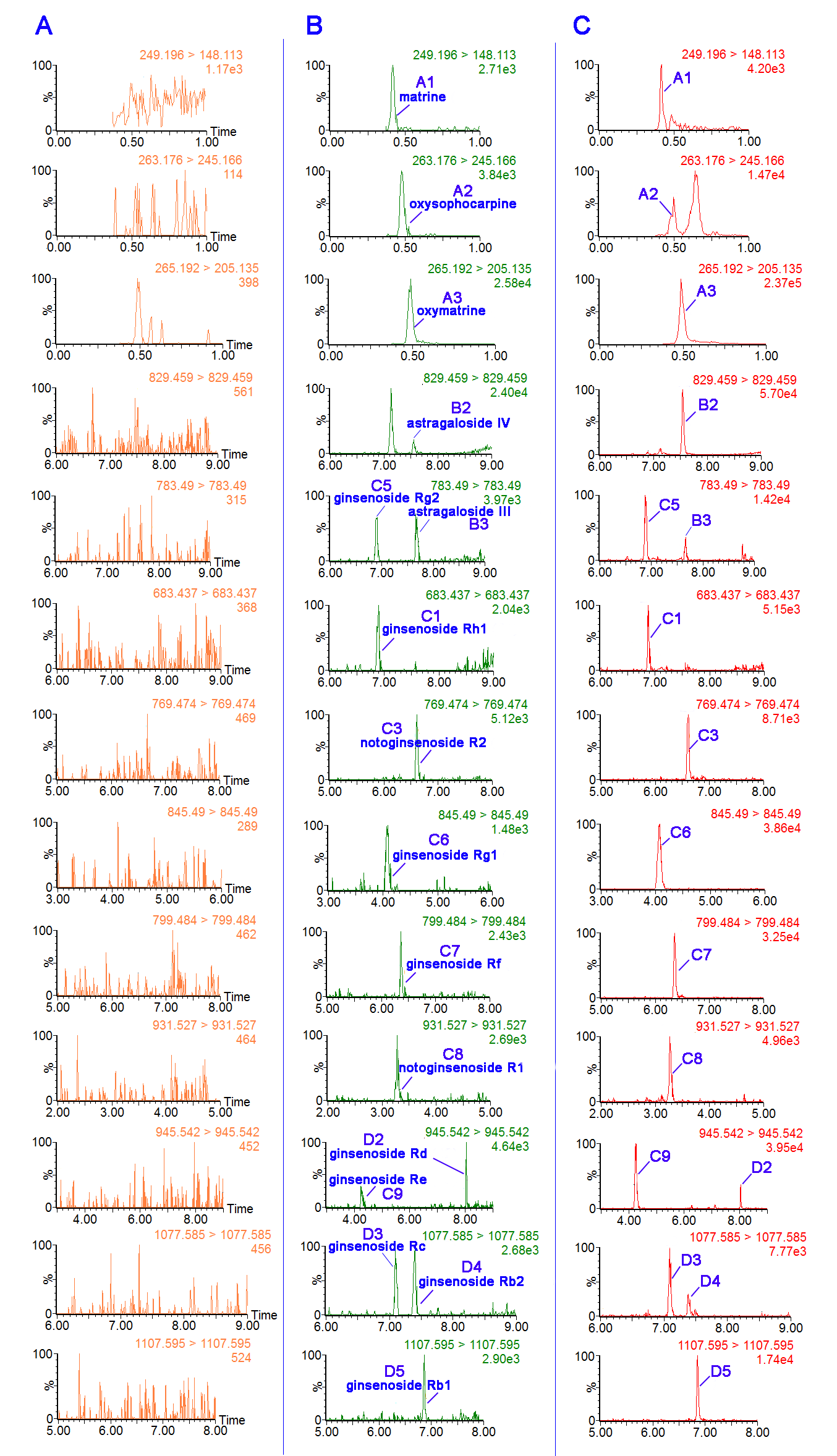


**Figure S4**





**Figure S5**
